# Characterization of S40-like proteins and their roles in response to environmental cues and leaf senescence in rice

**DOI:** 10.1186/s12870-019-1767-1

**Published:** 2019-05-02

**Authors:** Xiangzi Zheng, Muhammad Jehanzeb, Habiba, Yuanyuan Zhang, Li Li, Ying Miao

**Affiliations:** Fujian Provincial Key Laboratory of Plant Functional Biology, College of Life Sciences, Fujian Agriculture & Forestry University, Fuzhou, China

**Keywords:** S40 protein family, Leaf senescence, Rice, Expression profiling, Environmental cues

## Abstract

**Background:**

Senescence affects the quality and yield of plants by regulating different traits of plants. A few members of *S40* gene family, the barley *HvS40* and the *Arabidopsis AtS40–3*, have been shown to play a role in leaf senescence in Barley and *Arabidopsis*. Although we previously reported that S40 family exist in most of plants, up to now, no more function of S40 members in plant has been demonstrated. The aim of this study was to provide the senescence related information of *S40* gene family in rice as rice is a major crop that feeds about half of the human population in the world.

**Results:**

A total of 16 *OsS40* genes were identified from the genome database of *Oryza sativa L. japonica* by bioinformatics analysis. Phylogenetic analysis reveals that the 16 OsS40 proteins are classified into five groups, and 4 of the 16 members belong to group I to which also the HvS40 and AtS40–3 is assigned. *S40* genes of rice show high structural similarities, as 13 out of the 16 genes have no intron and the other 3 genes have only 1 or 2 introns. The expression patterns of *OsS40* genes were analyzed during natural as well as stress-induced leaf senescence in correspondence with senescence marker genes. We found that 6 of them displayed differential but clearly up-regulated transcript profiles under diverse situations of senescence, including darkness, nitrogen deficiency, hormone treatments as well as pathogen infection. Furthermore, three OsS40 proteins were identified as nuclear located proteins by transient protoplast transformation assay.

**Conclusions:**

Taking all findings together, we concluded that *OsS40–1*, *OsS40–2*, *OsS40–12* and *OsS40–14* genes have potential regulatory function of crosstalk among abiotic, biotic and developmental senescence in rice. Our results provide valuable baseline for functional validation studies of the rice S40 genes in rice leaf senescence.

**Electronic supplementary material:**

The online version of this article (10.1186/s12870-019-1767-1) contains supplementary material, which is available to authorized users.

## Background

Leaf senescence is an integral part of the final stages of plant development, and is controlled by a fine-tuned complex regulatory network [1]. Internal factors such as age of plants, hormones and external (a) biotic factors affects the regulation of leaf senescence [2]. Thousands of genes shows differential expression pattern at the onset and during the development of senescence [3, 4]. It has revealed a wide range changes in gene-expression during senescence in many plants such as *Arabidopsis* [4, 5], wheat [6], barley [7], rice [8] and aspen [9]. Senescence is a highly organized process that requires specific genes expression [10, 11] referred as senescence associated genes (SAGs) that account for 10% of a plant genome [3, 12]. Most of the SAGs are not only expressed during age dependent senescence, but are also expressed during stress induced senescence, such as wounding, darkness, desiccation, treatments of the leaves with hormones and in response to pathogen infections [2, 13]. These SAGs are activated by different transcription factors (TFs), among which NAC and WRKY are the two major groups that are involved in plant senescence [3]. Hence the expression of numerous SAGs can be influenced by the regulation of a specific gene that encodes a specific TF [14].

Despite the fact that senescence strongly influence yield of crop plants, only few SAGs had been isolated from crop plants such as wheat and barley [15], before the employment of microarrays for studies on wheat and barley leaf senescence [16, 17]. In barley, *HvS40* is shown to be senescence marker gene [18, 19]. The *HvS40* gene is up-regulated during natural senescence of barley primary leaves as well as dark induced senescence of detached leaves [18–21]. In a transcriptome study, the orthologous gene of wheat also showed enhanced expression during senescence of flag leaves [16]. Similarly in *Arabidopsis*, Seven of eleven genes, *AtS40–1*, *AtS40–2*, *AtS40–3*, *AtS40–4*, *AtS40–5*, *AtS40–6* and *AtS40–7* showed enhanced transcripts levels in senescent leaves compared to nonsenescent leaves [22]. The expression level of *AtS40–1*, *AtS40–2* and *AtS40–5* genes were induced after 2 days of darkness incubation while *AtS40–3* and *At-S40–4* showed increased expression only one day after darkness incubation [22]. Furthermore, *HvS40* shows enhanced expression in leaves treated with methyl jasmonate (MeJA), salicylic acid (SA) and infected by *Pyrenophora teres* [19]. Similarly, the expression levels of three genes *AtS40–2*, *AtS40–3* and *AtS40–4* were shown to be significantly increased after treatment of *Arabidopsis* plants with SA, abscisic acid (ABA) or pathogen *Pseudomonas syringae pv* for only 1 day [22]. Because of the nuclear localization of HvS40 protein, it is considered a candidate for regulating senescence related genes in the nucleus [19]. After transformation with onion epidermal cells, both the AtS40–3-GUS construct [22] and HvS40-GUS fusion construct showed similar GUS activity distribution in the nucleus [19].

The *Arabidopsis* T-DNA insertion mutant *s40–3a* showed delayed senescence compared to the wildtype [22]. The expression levels of *WRKY53* and *SAG12,* marker of early and late changes in gene expression during leaf senescence respectively, were clearly decreased in the *s40–3a* mutant compared to that in wildtype at all stages of senescence [22]. The overall expression analyses of *AtS40–3* gene in both the wildtype and mutants proposed that *AtS40–3* gene acts as an activator of downstream *WRKY53* and *SAG12*. Similarly, lower expression levels of two SAG markers, *SAG12* and *SEN1* [23], during dark condition in *s40–3a* mutant in comparison to that in wild type indicated that *AtS40–3* positively regulated senescence in both natural light as well as dark conditions [22]. Although we previously reported that S40 family exist in most of plants [24], up to now, no more information of S40 members in rice has been demonstrated.

In this study, we identified rice *S40* gene family consisting of 16 genes and analyzed their phylogeny, gene structure, chromosomal location, conserved motif determination, cis- acting elements and physical and chemical parameters prediction. Moreover, we comprehensively analyzed gene expression patterns of *OsS40* genes during natural as well as artificially (a) biotics stress induced senescence in correspondence with senescence marker genes using qRT-PCR. The subcellular localization of nine members including two genes showing overlapping expression profile among abiotic, biotic and developmental senescence are also identified. These findings can be used for further functional validation studies of the rice *S40* genes in rice leaf senescence.

## Results

### Identification and characterization of the rice *S40* genes

S40 belongs to DUF584 Family, it contains DUF584 domain, the sequence: GRXLKGR(D/E)(L/M)XXXR(D/N/T)X(I/V)XXXXG(F/I) is shared by all members belong to DUF584 group. The barley *HvS40* gene encodes a member of the DUF584 group gene while in *Arabidopsis*, fifteen proteins belong to this family that was classified into five groups based on similarities of their amino acid sequences [22]. To identify *S40* like genes in rice, HvS40 protein sequence was blasted as a query sequence against the rice genome. In total, sixteen similar hits were found in rice genome and these genes were tentatively named *OsS40–1* to *OsS40–16* (Table 1). The complete open reading frames (ORFs) of the retrieved *OsS40* genes ranged from 393 bp (*OsS40–2* and *OsS40–7*) to 780 bp (*OsS40–10*) (Table 1).

**Table 1:**
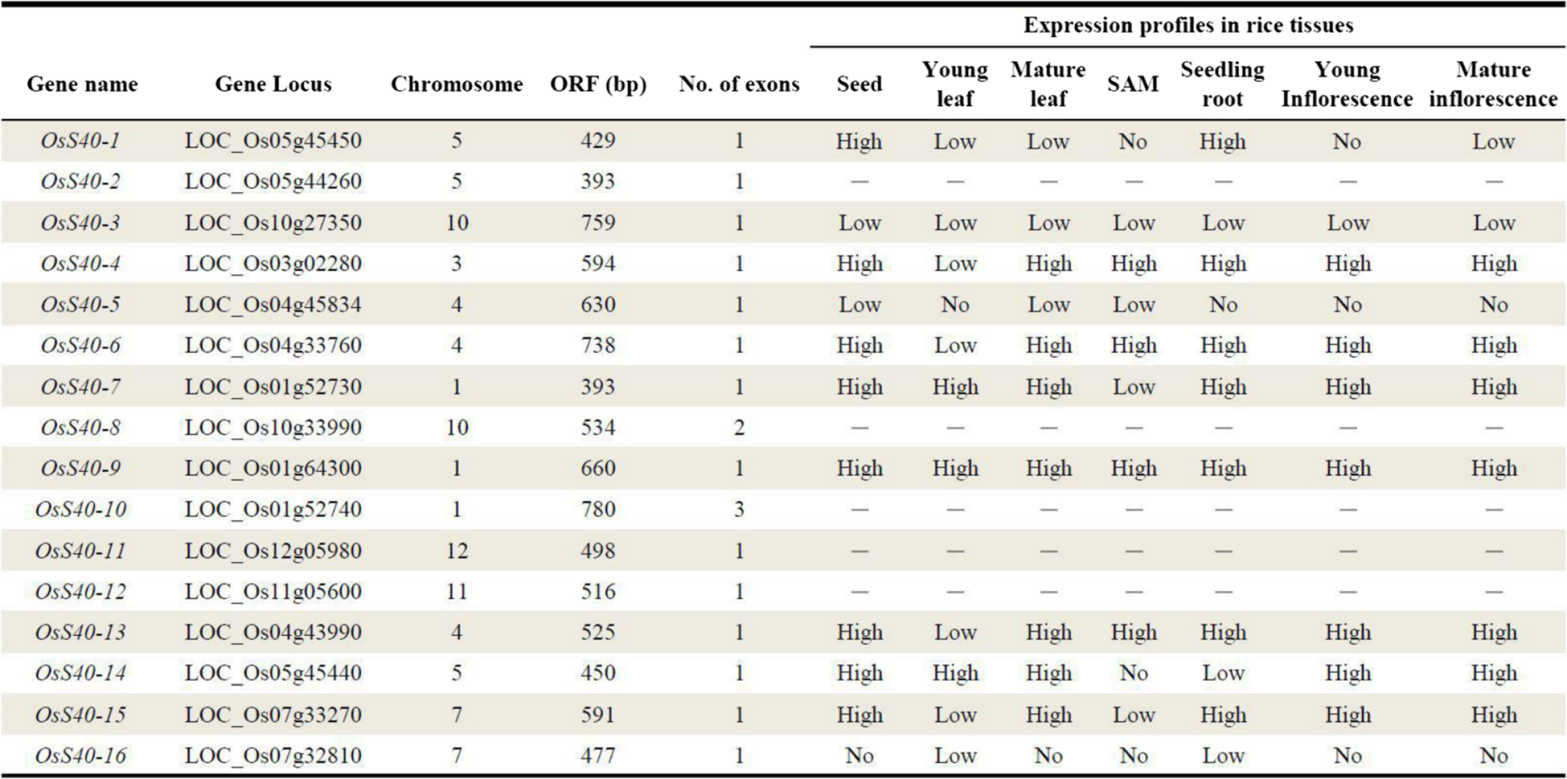
Overview of *S40* genes identified in rice genome and their predicted expression patterns in different tissues (http://bar.utoronto.ca/efprice/cgi-bin/efpWeb.cgi)

*ORF* open reading frame, *No.* number, *SAM* shoot apical meristem

To characterize the structural diversity of the OsS40 genes, exon/intron organization analysis of the individual OsS40 genes was performed. Number of exons and introns of *S40* genes in rice were calculated using Gene Structure Display Server database (http://gsds.cbi.pku.edu.cn/). *S40* genes of rice showed high structural similarity, as 13 out of the 16 genes have no introns while the other 3 genes have only 1 or 2 introns. Moreover, 5 genes, including *OsS40–2*, *OsS40–3*, *OsS40–8*, *OsS40–12* and *OsS40–16*, have no untranslated regions (UTR) (Additional file 1: Figure S1).

To gain insight into the organization of *S40* genes in rice, the chromosomal location of the *OsS40* genes was analyzed. Chromosome Map Tool from integrated rice science database (http://viewer.shigen.info/oryzavw/maptool/MapTool.do) was used to locate *OsS40* genes on chromosome. It revealed that 9 out of 16 *OsS40* genes are distributed among three chromosomes (Chr1, Chr4 and Chr5), while none of the genes are located on four chromosomes (Chr2, Chr6, Chr8 and Chr9). The other chromosomes contain one or two *S40* genes (Additional file 2: Figure S2). High-density *OsS40* gene clusters were mapped in certain chromosomal regions, e.g., in the proximal regions of Chr1 and Chr7 and in the distal regions of Chr4 and Chr5 (Additional file 2: Figure S2). These data may provide helpful information concerning the expansion of the rice *S40* gene family.

### Phylogenetic relationships of the rice *S40* genes

**Fig. 1:**
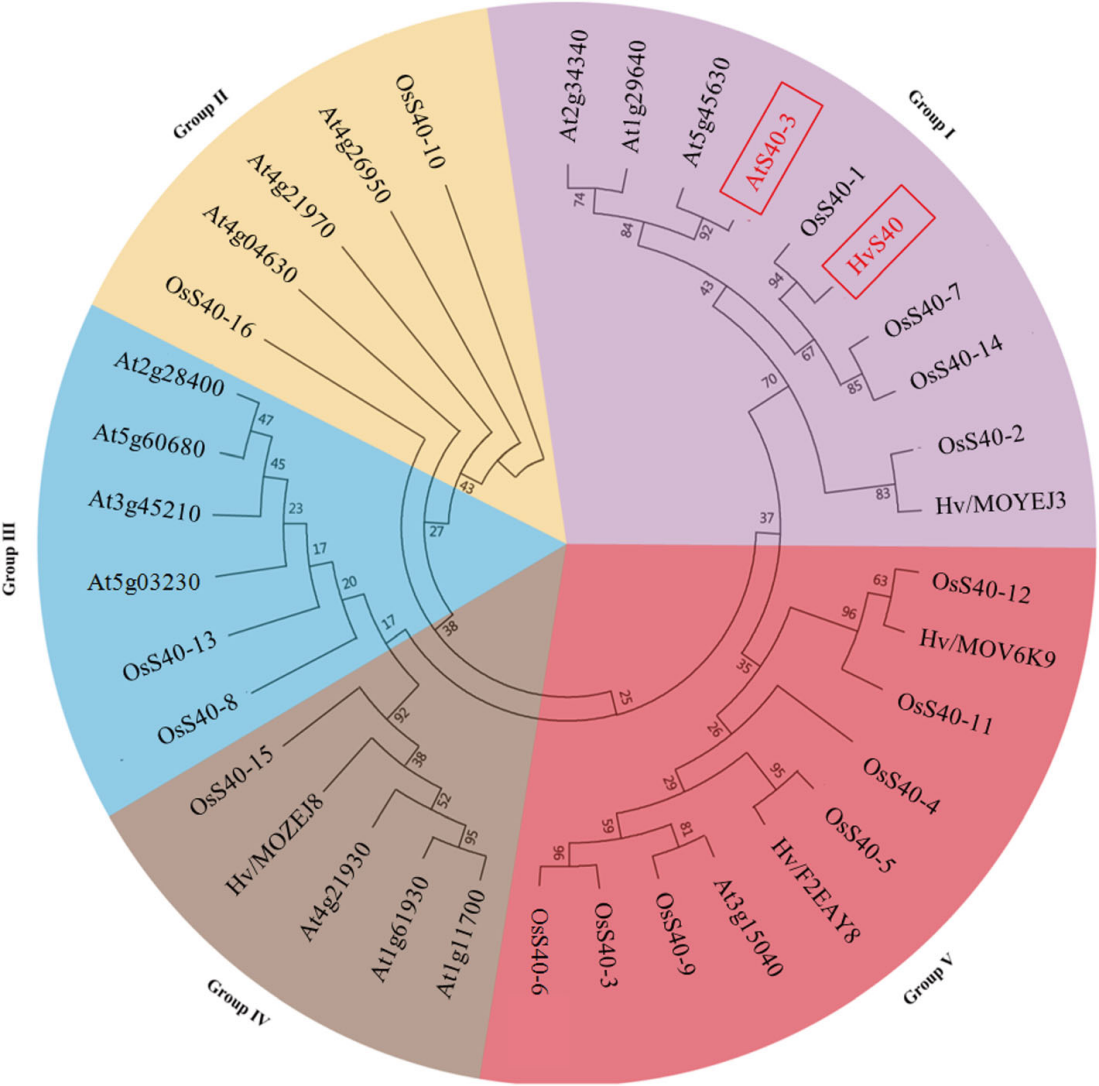
Phylogenetic analysis of the DUF584-family proteins from *Arabidopsis*, barley and rice. The un-rooted phylogenetic tree was constructed using MEGA 6.0 and the Neighbor-Joining method. The full length amino acid sequences were aligned using ClustalW. The bootstrap test was performed with 100 iterations. AT (*Arabidopsis*), Os (Rice) and Hv (Barley)

To survey the possible evolutionary relationships between S40 proteins from rice and previously reported S40 proteins from *Arabidopsis* and barley [22, 24], sequence alignments were performed and an un-rooted phylogenetic tress of 16 OsS40s, 15 AtS40s and 5 HvS40s was constructed using neighbor joining (NJ) method by MEGA 6.0 with 1000 bootstrap replicates. It indicated that the S40 family proteins of rice, *Arabidopsis* and barley could be classified into 5 groups (Fig. 1). 4 of the 16 DUF584 proteins of rice belong to group I to which also the HvS40 and AtS40 is assigned. From the further proteins of rice, 2 are present in group II and III each while only one protein appears in group IV. By contrast, 7 S40 proteins of rice cluster in group V, which contains only one *Arabidopsis* protein and two barley proteins (Fig. 1). No apparent similarity was identified by comparing amino acid sequences of the two S40 proteins of rice, four of *Arabidopsis* and one of barley from group I to the amino acid sequence of HvS40 protein. However, all the compared protein sequences shared the conserved DUF584 domain sequence (Additional file 2: Figure S3).

The phylogenetic relationship and classification of S40 members in rice were further supported by motifs analysis. Ten individual motifs, designated motif 1 to 10, for 16 rice S40 proteins, AtS40–3 and HvS40 were sensitized using MEME software (Additional file 2: Figure S4). As predicted, DUF584 domain corresponded to motif 1, whereas the functions of the other putative motifs were elusive since they lacked homologs in protein motif database SMART or Pfam. Motifs 1, 2, 3 and 4 were present in all these proteins while other motifs have specific distributions among different proteins. Motif 8 or motif 9 was clade-specifically distributed in protein members that belong to group I. Furthermore, OsS40–2 and HvS40 showed similar motif distribution patterns. AtS40–3 is unique in motif distribution patterns because of the presence of motif 9 and the most similar rice S40 proteins to AtS40–3 are OsS40–16 and OsS40–14 (Additional file 2: Figure S4), implying functional similarities among these S40 members.

### Cis elements comparison in the promoters of *HvS40, AtS40–3* and *OsS40* genes

To uncover *cis* elements that might be involved in the initiation of *S40* gene transcription during senescence and to explore elements related to developmental senescence, the promoter of the *S40* genes in rice was revealed using PlantCARE database (http://bioinformatics.psb.ugent.be/webtools/plantcare/html/) and compared with the promoters of *AtS40–3* and *HvS40* [25]. Except *OsS40–11*, the upstream of *OsS40* genes as well as *HvS40* and *AtS40–3* genes showed the presence of W-Boxes (C/T)TGAC(T/C), the elements known to be the binding site of WRKY TFs [26]. In addition, many MYC (MYC TF recognition sites [27]), MYB (MYB TF recognition sites [27]) and Dof (Dof proteins core binding sites [28] were found in the promoters of all rice *S40* genes as well as *HvS40* and *AtS40–3* (Additional file 2: Table S1), suggesting that the promoters of S40 genes might be targeted by different TFs. Abundant CGCG boxes (Motifs recognized by calmodulin-binding proteins, which involved in multiples signaling pathways in plants [29]) and several ABA-responsive elements (ABRE) recognition sequences [30] were identified in promoter regions of some rice *S40* genes while no CGCG box region was found in *AtS40–3* gene promoter. Moreover, a few dehydration-responsive elements/C-repeats (DRE/CRT) [31] and low-temperature responsive elements (LTRE) [32] were synchronously present in the promoters of 8 *OsS40* genes as well as *HvS40* while only one LTRE appeared in AtS40–3 promoter (Additional file 2: Table S1). This information implied that transcripts of rice *S40* genes might be affected by different environmental and developmental conditions, as has been shown in the study of *HvS40* [19, 33, 34]. In potato, a single-strand DNA binding factor, StWHIRLY1, was shown to bind to the elicitor response elements (ELRE) identified in the promoter of *PR10a* [35, 36]*.* Recently, it has been demonstrated that two ELRE elements in the promoter region of *HvS40* gene can interact with HvWHIRLY1 [33]. However, ELRE elements were rarely observed in promoter sequences of *OsS40* genes as only one ELRE was found in the promoters of *OsS40–7*, *OsS40–13*, *OsS40–15* and *OsS40–16* (Additional file 2: Table S1).

### Expression profiles of *OsS40* candidate genes during natural senescence

Generally, plants grown under ideal conditions will undergo natural senescence, which is primarily controlled developmentally. To obtain clues regarding possible functions of *OsS40* genes in different tissues during plant development of rice, an overview of expression changes of these *OsS40* candidates in seed, root, shoot apical meristem (SAM), developing leaves as well as inflorescence was obtained from the rice eFP Browser (http://bar.utoronto.ca/efprice/cgi-bin/efpWeb.cgi) [37]. Although transcript data of some *OsS40* members is not available from the database, the remaining rice *S40* genes displayed differential expression patterns in these organs (Table 1). *OsS40–9* looked highly expressed throughout rice development. On the contrary, *OsS40–3*, *OsS40–5* and *OsS40–16* transcripts were low or undetectable in all these tissues examined. It was noted that the transcript value of *OsS40–4*, *OsS40–6*, *OsS40–13* and *OsS4–15* was low in young leaf, but high in mature leaf (Table 1), implying their involvement in natural development of rice leaf.

**Fig. 2:**
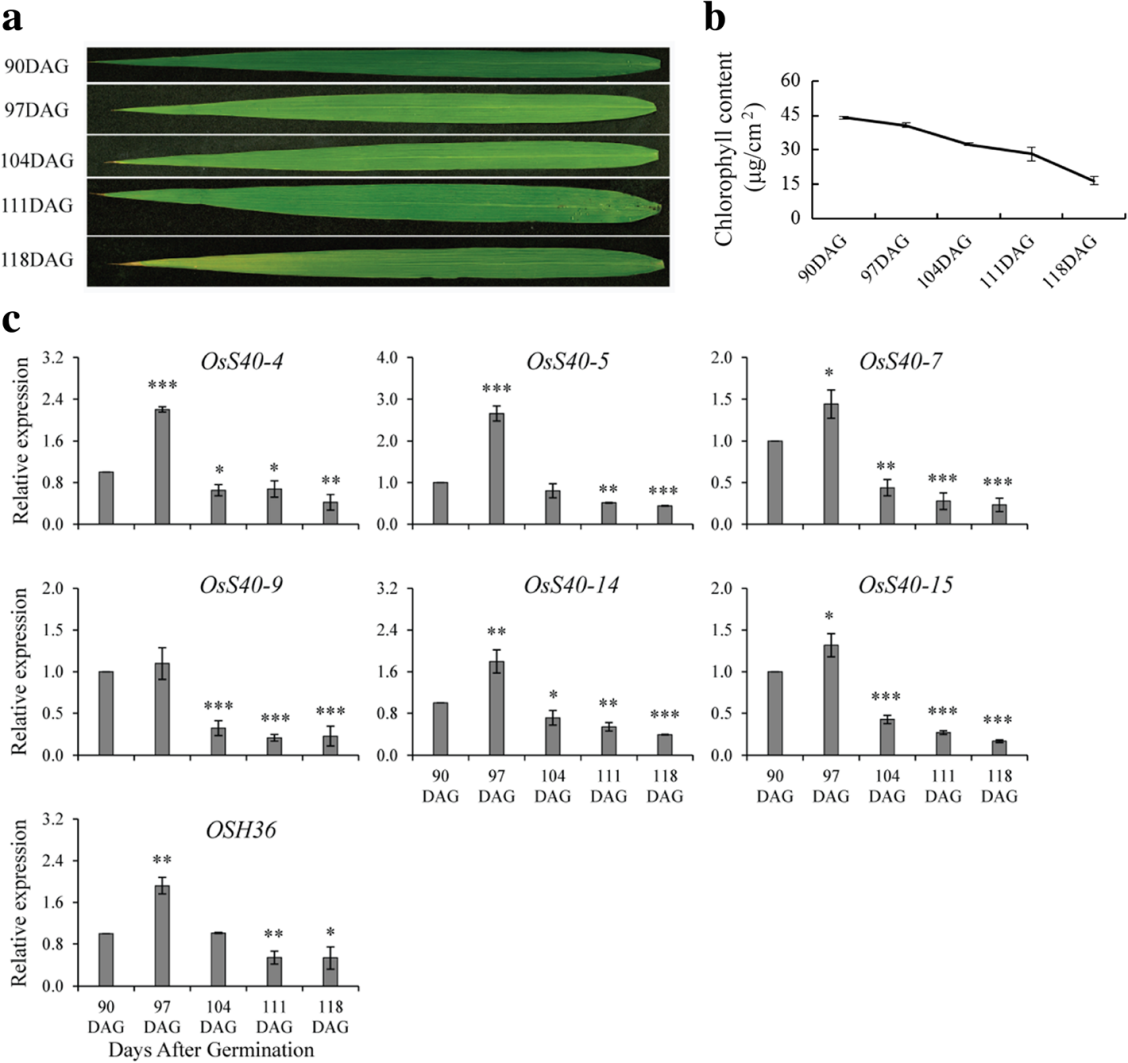
Expression analysis of *OsS40* genes during natural senescence of flag leaves. **a** Phenotype of rice flag leaves collected at different late development stages (90DAG, 97DAG, 104DAG, 111DAG, 118DAG). DAG, Days after germination. **b** Development dependent alteration of chlorophyll content in rice flag leaves. **c** qRT-PCR analyses of the six differentially expressed *OsS40* genes along with senescence marker gene, *OSH36*. Transcript levels are expressed relative to that of rice actin in each sample, and values are mean ± S.D. Student t-test was used to generate *P* value. *P ≤ 0.05, **P ≤ 0.01 and ***P ≤ 0.001

Rice flag leaf is proposed to serve as a determinant for grain yield, since flag leaves not only supply the seeds carbon components by photosynthesis but also transport the useful nutrients from senescent leaves into young panicles during the grain-filling period [38, 39]. Characterization of *OsS40* genes during natural senescence of flag leaves would be more valuable for understanding the mechanisms of flag leaf senescence and favoring grain yield. For collecting the senescent flag leaves, rice plants were grown to the ripening stage (4 months). The plants were keenly observed for their phenotypic changes, as yellowing color is one of the striking signals of senescence. The yellowish phenotype was observed at 90th day after germination (DAG) and on the same day, samples were collected as the onset of senescence. Furthermore, samples were collected for four times with some interval, at the onset of senescence: 90DAG, 97DAG, 104DAG, 111DAG, 118DAG (Fig. 2 a, b). To determine transcript profiles of rice S40 genes at different late development stages of flag leaves, primers were designed for all the sixteen members and used for RT-PCR. Six of the sixteen genes, *OsS40–4, OsS40–5, OsS407, OsS40–9, OsS40–14* and *OsS40–15* showed differential expression level during semi quantitative RT-PCR analysis (Additional file 2: Figure S5). The results were confirmed by qRT-PCR (Fig. 2c). Among the six altered expressed genes, three belong to group V, two belong to group I and one is the single group IV gene. The gene expression tendency showed increased expression level at early senescence to decreased expression level at late senescent leaves. One of the senescence associated genes, *OSH36* [40], also showed the same expression pattern as a positive control. Overall, our results demonstrated that these six members of the *OsS40* family showed enhanced expression at the onset of senescence while showed a decreased expression pattern at later stage of senescence.

### Expression profiles of *OsS40* candidate genes during nitrogen stress

**Fig. 3:**
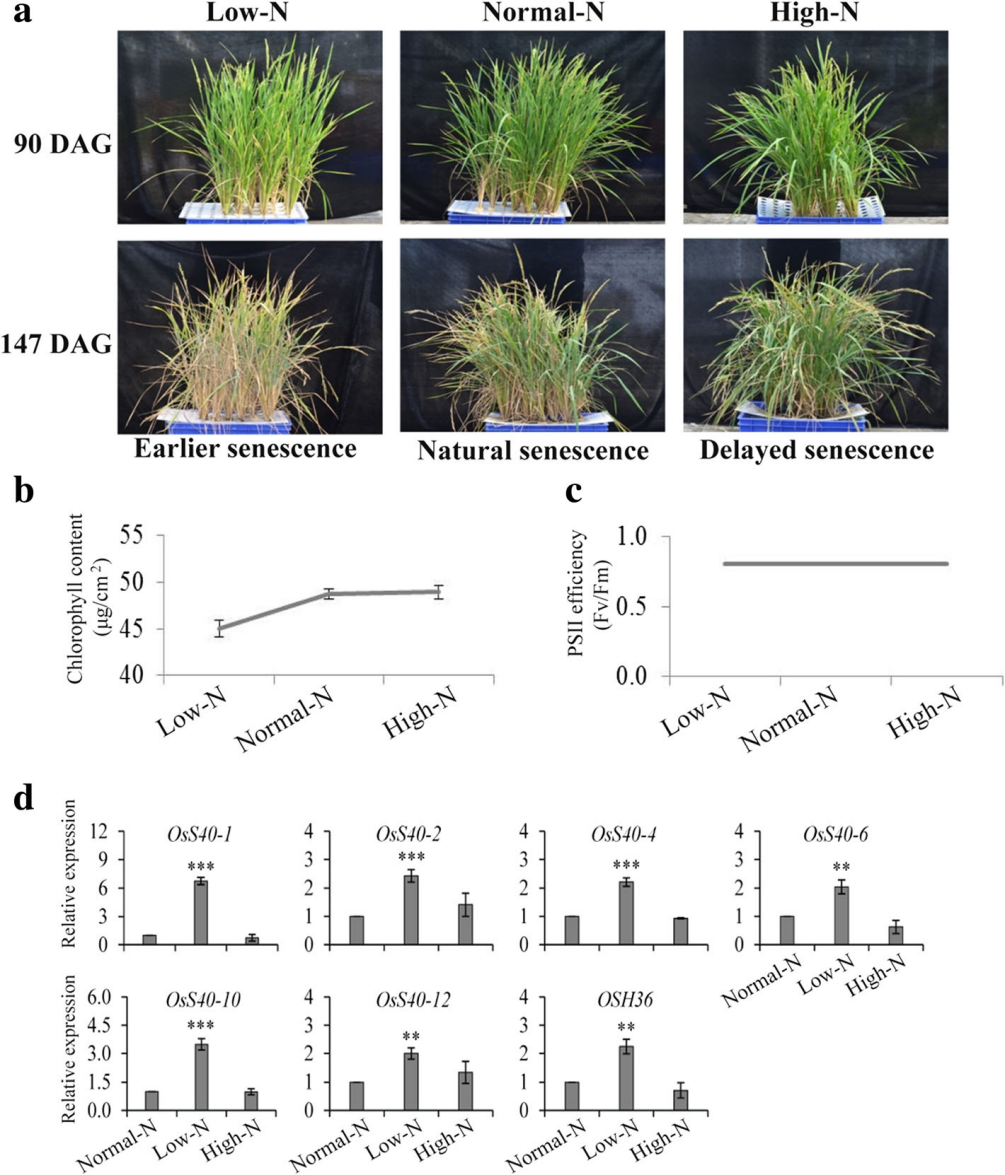
Expression analysis of *OsS40* genes in rice plants growing under different nitrogen concentrations. **a** Phenotypes of 13 weeks (90DAG) and 21 weeks (147DAG) old rice plants growing under normal nitrogen (Normal-N) as a control, low nitrogen (Low-N) or high nitrogen concentration (High-N). The nitrogen nutrient was applied to the growth media of growing seedlings during the entire development. **b and c** Plants were characterized by the chlorophyll content (μg/cm2) and relative photochemical efficiency of photosystem II (Fv/Fm) of entire rosettes. The error bars indicate the standard error of ten independent measurements for chlorophyll content and five independent measurements for chlorophyll photosystem II efficiency. **d** Quantitative real-time PCR analysis of the six differentially expressed *OsS40* genes along with senescence marker *OSH36* gene. Transcript levels are expressed relative to that of rice actin in each sample, and values are mean ± S.D. Student t-test was used to generate *P* value. *P ≤ 0.05, **P ≤ 0.01 and ***P ≤ 0.001

Nutrient deficiency, in particular the limitation of nitrogen (N), has been proved to be capable to accelerate plant senescence. Early leaf senescence under low-N supply and delayed leaf senescence upon surplus-N supply has been demonstrated by several reports [41–43]. Precocious flag leaf senescence caused by nitrogen starvation can result in yield decrease [44]. Thus we examined the expression levels of these *OsS40* genes in senescent flag leaves of rice growing under low-N or surplus-N conditions. To test the effect of altered N availability on rice development, an experimental condition was designed in which hydroponic culture system was used with only roots submerged in growth medium. Rice plants were separately cultured in three different types of growth media containing normal nitrogen supply (Normal-N) used as a control, half of the normal nitrogen supply (Low-N) or double of the normal nitrogen supply (High-N) compared to control. Rice plants grown in the medium with deficient N availability showed earlier senescence, while plants with excessive supply of N showed delayed senescence compared to the control plants (Fig. 3a). The relative photochemical efficiency of photosystem II (Fv/Fm) of entire rosettes was similar in all types of plants while the chlorophyll content were decreased in lower nitrogen supplied plants compared to normal nitrogen supplied plants (Fig. 3b, c).

To identify *OsS40* genes with common and distinct expression patterns in response to altered N supply, flag leaves were collected at 90DAG from rice plants growing in all the three types of media and gene expressions were analyzed via semi qRT-PCR at first. Nine out of the sixteen *OsS40* genes showed detectable expression in the control sample (Normal-N) and 6 among them, *OsS40–1, OsS40–2, OsS40–4, OsS40–6, OsS40–10* and *OsS40–12,* displayed differential expression upon changed N supply (Additional file 2: Figure S6). These 6 differentially expressed genes were further analyzed by qRT-PCR that confirmed the enhanced transcript levels of these six genes. However, the expressions of all these six genes were up-regulated in plants supplied with low N compared to control (Normal-N), while their expression patterns were similar between control and high N supply (Fig. 3c), suggesting these six *OsS40* members may take part in the early senescence of flag leaves caused by N limitation, but not participate in the delayed senescence induced by surplus N supply. Two of the six up-regulated genes, *OsS40–1* and *OsS40–2,* belong to group I to which *HvS40* in Barley [19] and *AtS40–3* in *Arabidopsis* [22] also belong. *OSH36* [40], as a positive control, also showed the similar expression pattern .

### Expression profiles of *OsS40* candidate genes in response to darkness treatment

**Fig. 4:**
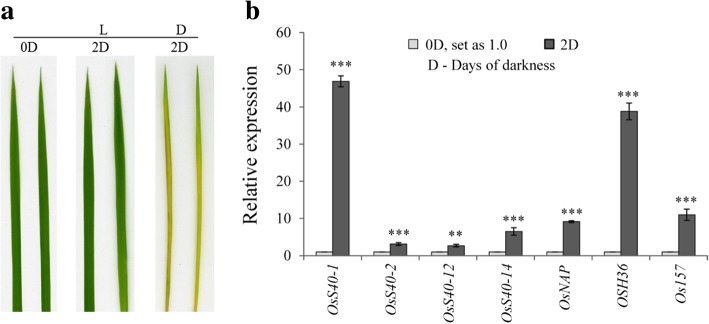
Expression analysis of *OsS40* genes in response to dark treatment. **a** Phenotype of detached rice leaves after darkness treatment. Detached leaves from 4-week-old rice seedlings were incubated in the deionized water in darkness for 2 days (D). As a control, the detached leaves were incubated with water at the same time in a light/dark regime (L). **b** qRT-PCR analysis of the four differentially expressed *OsS40* genes along with senescence marker genes, *OsNAP*, *OSH36* and *Os157,* after darkness treatment. Transcript levels are expressed relative to that of rice *actin* in each sample, and values are mean ± S.D. Student t-test was used to generate *P* value. *P ≤ 0.05, **P ≤ 0.01 and ***P ≤ 0.001

Chlorophyll degradation and protein catabolism are some typical symptoms of senescence process. Dark-induced senescence has frequently been used as a model system to promote these symptoms to study natural senescence in plants [13, 45]. The barley *HvS40* was first identified as a SAG due to its elevated mRNA level during darkness-induced senescence of detached leaves [18]. To explore which of the OsS40 members belong to dark-induced SAGs, these candidate genes were tested for expression after darkness treatment. When the detached rice leaves floating on water were incubated in darkness for 2 days, a visible yellowing phenotype was observed on the treated leaves but not on the control leaves (Fig. 4a), indicating that the accelerated senescence process occurred after dark incubation. Via semi qRT-PCR, four of the sixteen genes, *OsS40–1*, *OsS40–2*, *OsS40–12* and *OsS40–14*, showed upegulated expression levels in the leaves exposed to darkness for 2 days (Additional file 2: Figure S7). Analysis by qRT-PCR further confirmed the enhanced transcript levels of these four genes as well as the dramatic induction of several senescence marker genes, including *OsNAP, OSH36* and *Os157*, in the dark-treated leaves (Fig. 4b). This result indicated that the four OsS40 members are possibly implicated in dark-induced leaf senescence.

### Expression profiles of *OsS40* candidate genes in response to hormones

**Fig. 5:**
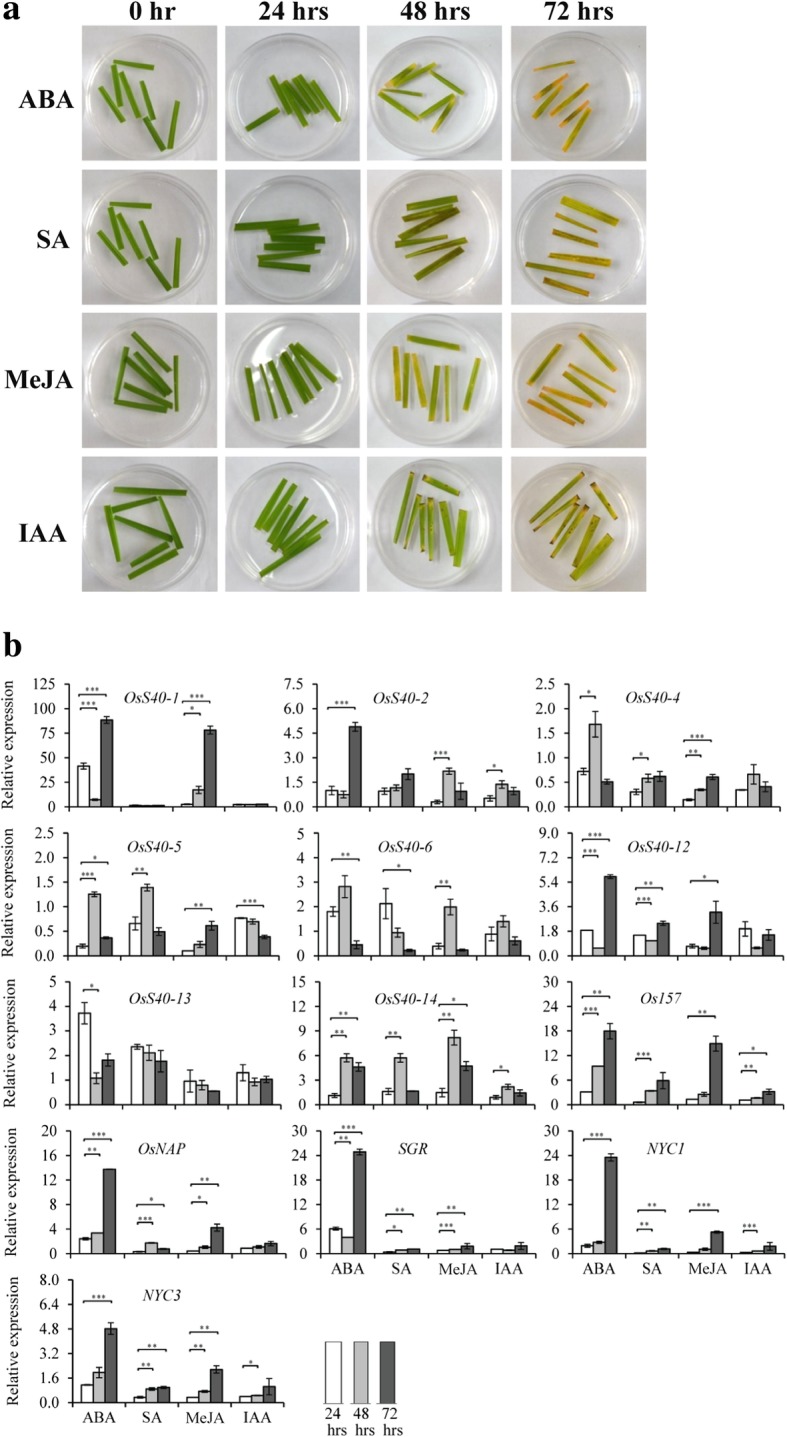
Expression analysis of *OsS40* genes under different hormone treatments. **a** Phenotypes of detached leaves from four weeks old rice plants under different hormone treatments. Photos of detached leaves floating on 200 μM concentration of ABA, SA, MeJa or IAA solution were taken at 0 h, 24 h, 48 h and 72 h. **b** Quantitative real-time PCR analyses of the eight differentially expressed *OsS40* genes along with senescence marker genes, *OSNAP*, *SGR*, *NYC1*, *NYC3* and *OS157*, upon ABA, SA, MeJA or IAA treatment. Transcript levels are expressed relative to that of rice *actin* in each sample, and values are mean ± S.D. Student t-test was used to generate *P* value. *P ≤ 0.05, **P ≤ 0.01 and ***P ≤ 0.001

Phytohormones like JA, SA, ABA, and ethylene has been described as inducers of leaf senescence, while gibberellic acid, cytokinins and auxin result in delayed leaf senescence [46, 47]. To test the influence of hormones on rice leaf senescence, mature leaves from 4 weeks old plants were harvested by cutting at the approximate middle of the petioles with a sharp scalpel to minimize wounding effects. The detached leaves were then floated in different concentrations (50 μM, 100 μM, 200 μM) of ABA, SA, MeJA or IAA for 0, 24, 48 and 72 h (hrs) to examine the effect of different doses of hormones and the time points were noted. Compared to water treatment, a clear promotion of yellowing was observed on leaves when incubated for 48 h floating on the four 200 μM hormone solutions (Fig. 5a and Additional file 2: Figure S8), suggesting that leaf senescence was significantly accelerated upon these hormone treatments. To identify the effect of different hormones on expression patterns of *OsS40* genes, samples were collected after 24 h, 48 h and 72 h of 200 μM concentration of hormones treatment and gene expressions were analyzed via semi qRT-PCR at first. Except *OsS0–16*, all the *OsS40* members showed detectable expression in this experiment and eight among them presented altered transcript levels after treatment with different hormones (Additional file 2: Figure S9), which was further determined by qRT-PCR analysis.

Nearly half of the 16 members, *OsS40–1*, *OsS40–2*, *OsS40–4*, *OsS40–5, OsS40–6, OsS40–12* and *OsS40–14*, displayed enhanced expression patterns in the detached leaves treated with ABA for 48 h or 72 h, while *OsS40–13* was only highest expressed at 24 h after ABA treatment, which may be the early onset of ABA-triggered leaf yellowing (Fig. 5b). After SA treatment, mRNA levels of *Os40–4, OsS40–5, OsS40–12* and *OsS40–14* were moderately elevated at 48 h or 72 h, whereas *OsS40–6* was highly induced at 24 h, maybe the onset of SA-elicited leaf senescence (Fig. 5b). It is noteworthy that the seven *OsS40* genes strongly responsive to ABA treatment were also markedly up-regulated after exposure to MeJA (Fig. 5b), suggesting that these seven members may play redundant function in the common molecular mechanisms shared by ABA- and MeJA-mediated leaf senescence. As a control, the expressions of several SAG markers, such as *OsNAP* [48], *SGR* [49], *NYC1* [50], *NYC3* [51] and *Os157* [40] were also extremely induced upon ABA, SA or MeJA treatments (Fig. 5b). In contrast, only a few genes, *OsS40–2*, *OsS40–5* and *OsS40–14*, showed accumulated expression levels in respond to IAA, implying that most of OsS40 genes may be not important for IAA-induced leaf senescence. IAA is a biologically active auxin and several lines of evidence support the negative role of auxin in leaf senescence [52–54]. However, it has also been published that overexpressing an auxin-responsive gene, *SAUR36*, in *Arabidopsis* displayed an early leaf senescence phenotype [55] and auxin signaling pathway is required for a senescence-associated receptor kinase (SARK) mediated early leaf senescence [56]. Therefore, auxin may also play a promoting role in leaf senescence. Taken together our results (summarized in Table 2) suggest that some of the OsS40 genes may fulfill key roles in crosstalk among multiple hormone-dependent senescence pathways.

**Table 2:**
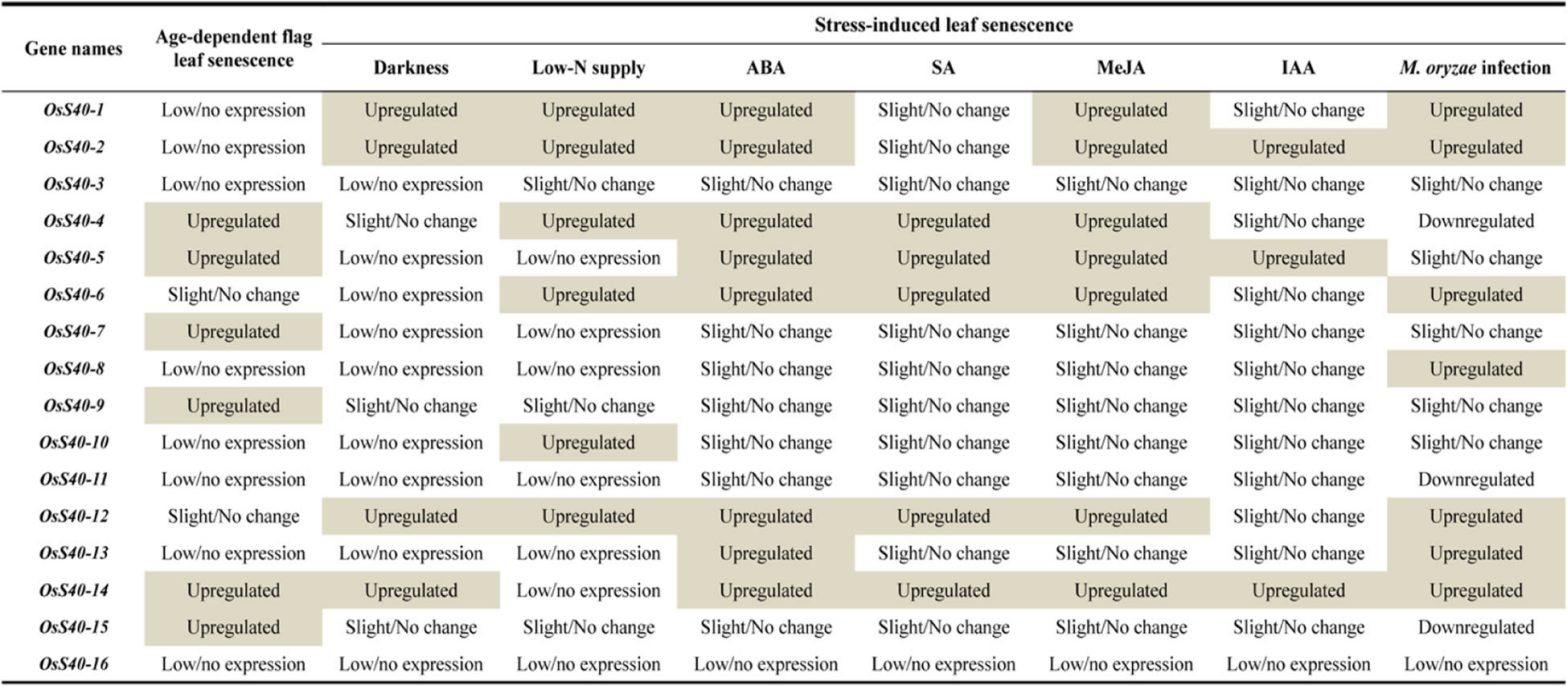
Summary of expression profiles of *OsS40* genes during natural senescence of flag leaves and in respond to various environmental stresses

### Expression profiles of *OsS40* candidate genes in response to pathogen infection

**Fig. 6:**
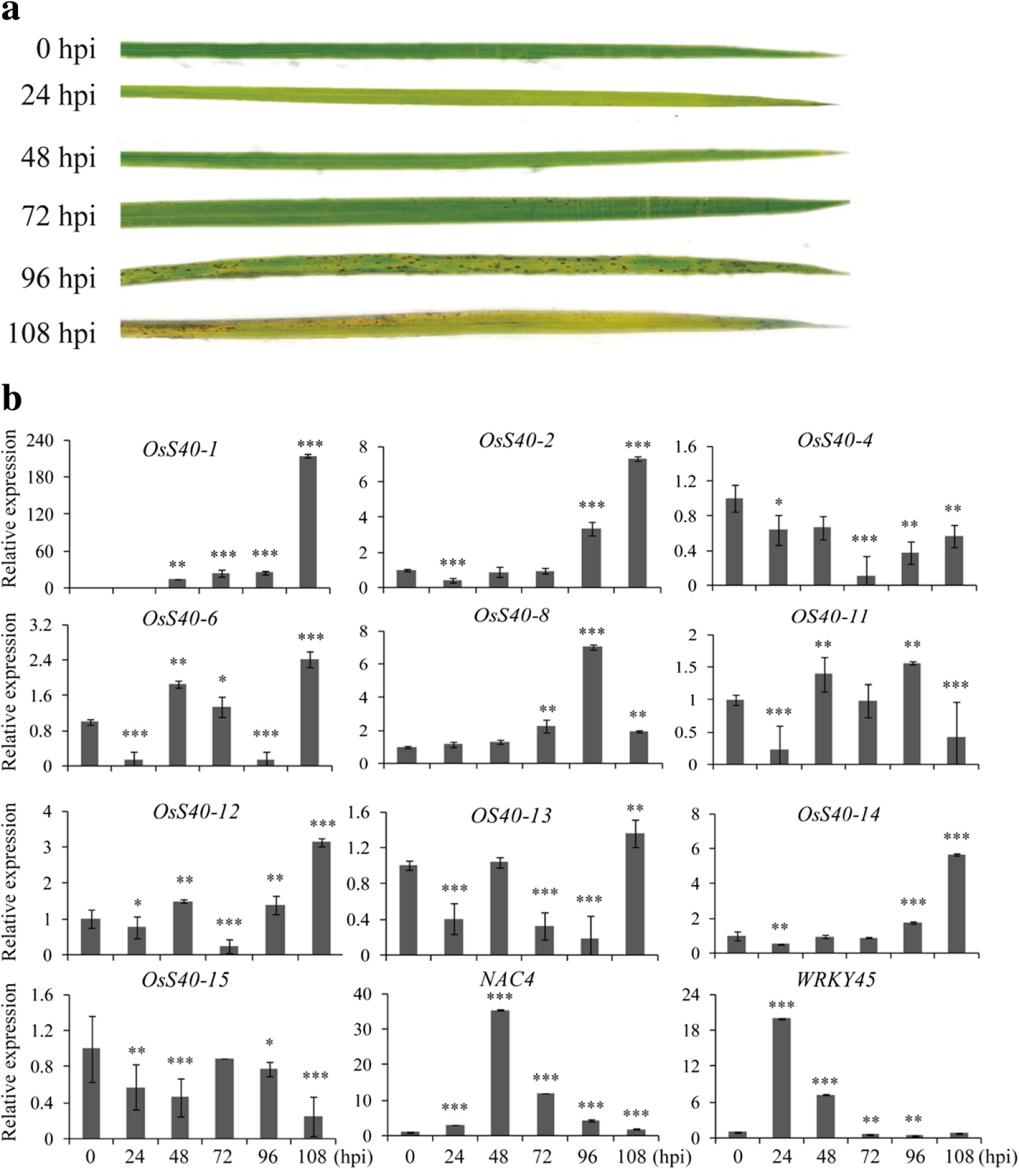
Expression analysis of *OsS40* genes in response to *M. oryzae* infection. **a** Phenotype of the leaves from 3 to 4 leaf stage of rice seedlings infected by *M. oryzae* strain Guy11. The infected leaves were excised and scanned at 24 hpi, 48 hpi, 72 hpi, 96 hpi and 108hpi. As a control the rice seedlings were sprayed with the 0.02% (*w*/*v*) Tween 20 solution only (Mock). hpi, hours post-inoculation. **b** qRT-PCR analysis of the ten differentially expressed *OsS40* genes along with the defense-related marker genes, *NAC4* and *WRKY45,* for pathogen treatment. Transcript levels are expressed relative to that of rice *actin* in each sample, and values are mean ± S.D. Student t-test was used to generate P value

Many phytohormones, especially SA and JA, have been demonstrated to contribute to systemic-acquired resistance in plant [57, 58]. Several members of *OsS40* gene family showed enhanced expression level in response to SA or MeJA, suggesting that they may play defense-related function during plant-pathogen interaction. To explore the expression of the *S40* candidate genes of rice upon infection with fungal pathogen, 3–4 leaf stage of rice seedlings were inoculated by spraying spores of *Magnaporthe oryzae* strain Guy11. As a control, the rice seedlings were sprayed with the 0.02%(*w*/*v*) Tween 20 solution only. The infected leaves were collected every 24 h until disease symptoms were clearly visualized at 108 h post inoculation (hpi) (Fig. 6a). To control the effect of the pathogen, transcript levels of two defense-related marker genes, *NAC4* [59] and *WRKY45* [60], were also measured during infection with fungus. As anticipated, they both showed a strong induction in the infected rice leaves.

Semi qRT-PCR analysis revealed that 10 out of the 16 genes displayed altered gene expression after treatment of the plants with the fungal pathogen (Additional file 2: Figure S10), which was further confirmed by qRT-PCR analysis. The five genes, *OsS40–1*, *OsS40–2*, *OsS40–6*, *OsS40–12* and *OsS40–14* that were responsive to SA or MeJA treatment, also had elevated mRNA levels in plants infected with *M.oryzae*. And they all were exclusively highly expressed at the late stage of the fungus infection on rice, when the infected leaves had developed necrotic lesions (Fig. 6 a and b). *HvS40* was also reported to have great mRNA accumulation only at the sites of infection with *Pyrenophora teres* [19]. It is speculated that these five *OsS40* genes and *HvS40* might be associated with the rapid senescence and cell death caused by necrosis. Additionally, increased transcript level after the infection also appeared in *OsS40–8* and *OsS40–13*, whose expressions were not affected by all the aforementioned treatments. However*, OsS40–4*, *OsS40–11* and *OsS40–15* showed decreased expression levels in the infected rice leaves (Fig. 6b), implying that they may play distinct or negative roles in the senescence triggered by pathogen infection.

### Subcellular localization of OsS40 proteins in rice cells

Information’s about all these sixteen S40 proteins such as, molecular weight (MW), number of amino acid, theoretical isoionic point (pI), instability index, aliphatic index and GRAVY (Grand Average of Hydropathy) were predicted by Protparam program (http://web.expasy.org/protparam/) (Additional file 2: Table S2). The value of isoionic point (pI) varied from 5.36 (OsS40–16) to 11.27 (OsS40–11). The corresponding molecular weight varied from 14.14 kDa (OsS40–7) to 28.52 KDa (OsS40–10). The molecular weights ranged from with an average of 19.67 kDa. Among these proteins, all had an unstable structure except *OsS40–13* with the instability index to be 27.77. OsS40–16 was the unstable one with an instability index of 89.11. All the S40 proteins in rice were found to be hydrophilic proteins with hydrophobicity score (GRAVY) below 0 (Additional file 2: Table S2).

**Fig. 7:**
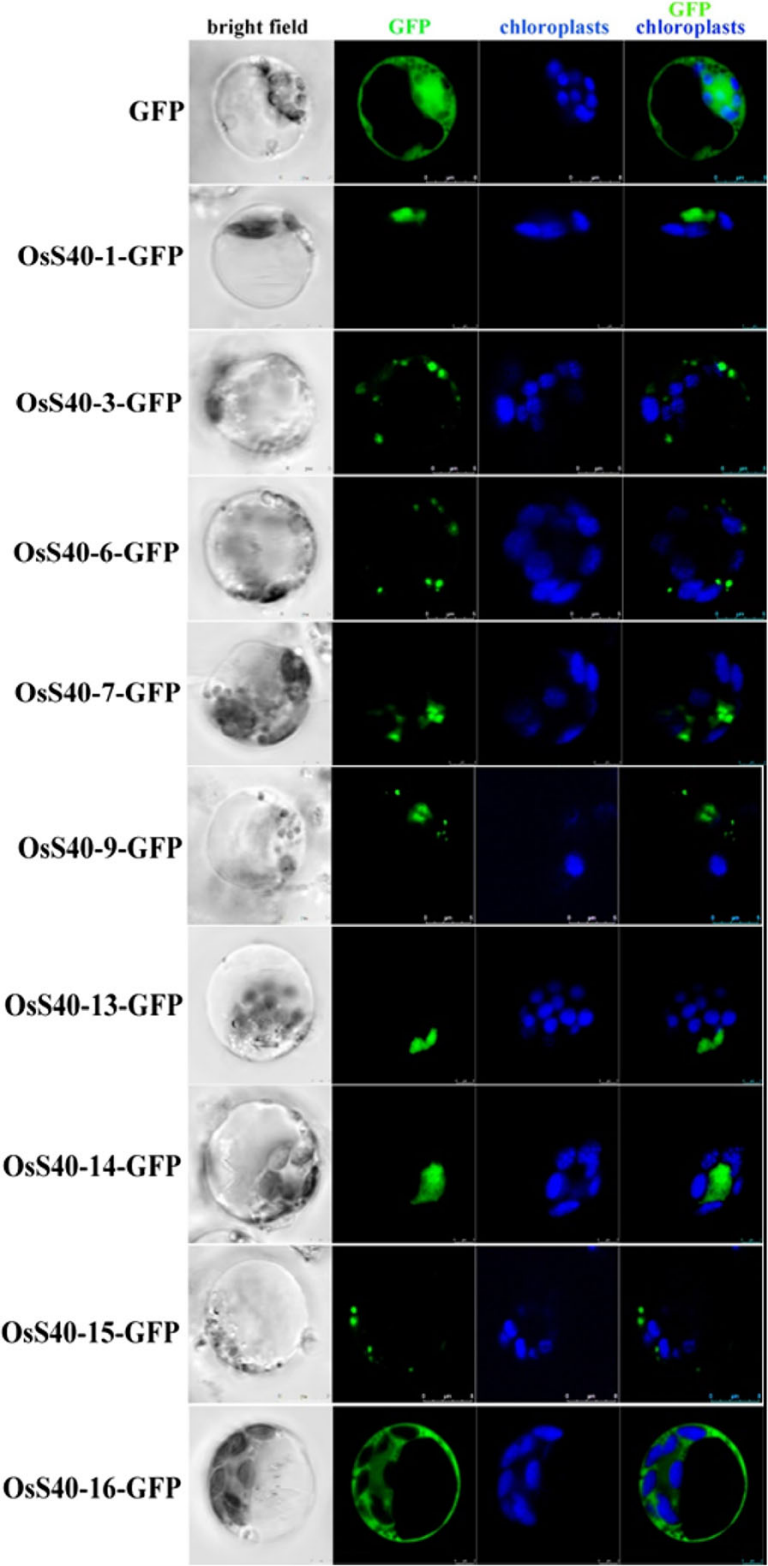
Subcellular localization of OsS40 proteins. Rice protoplasts transiently expressing OsS40s-GFP fusion protein were observed using confocal laser scanning microscope after 12 h transformation. The image shows representative optical sections of bright field and merged fluorescence of GFP (green) and chloroplast (blue) in protoplasts. The right column depicted the merged images

In order to investigate the subcellular localization of OsS40 members in rice cells, their coding regions were fused to the *GFP* gene and put under the control of the 35S CaMV-promoter. Isolated protoplasts from rice seedlings were transformed with these constructs by PEG-mediated transformation. In protoplasts, the expressed OsS40–1-GFP, OsS40–13-GFP and OsS40–14-GFP were efficiently accumulated in the nucleus while OsS40–16-GFP was distributed in the cytoplasm. On the other hand, OsS40–3-GFP OsS40–6-GFP OsS40–7-GFP OsS40–9-GFP and OsS40–15-GFP fluorescence were appeared as several speckles in cytoplasm (Fig. 7). Immunodectection experiments confirmed the expression and stability of these OsS40 members in rice cells (Additional file 2: Figure S11).

## Discussion

Several studies have presented that *S40* genes are related to plant senescence caused by aging or environmental changes, such as barely *HvS40* and *Arabidopsis AtS40–3* [19, 22, 34]. Genome-wide survey and alignment also showed S40 gene families exist in many plants [24]. In the present study, 16 DUF584-containing members were isolated based on HvS40 protein sequence and further characterized for their expression patterns during senescence as well as their localizations in rice cells. This screening investigation revealed that 6 of the 16 members are in respond to age-dependent or stress-induced leaf senescence. Furthermore, 2 of these candidates, OsS40–1 and OsS40–14, are mainly enriched in nucleus by transient expression in rice protoplast, which is similar to the subcellular distribution patterns of HvS40 and AtS40–3 [19, 22].

In analogy to *Arabidopsis* S40 family, the 16 OsS40 members are divided into 5 groups and their genes are unevenly interspersed on eight rice chromosomes (Fig. 1, Additional file 2: Figure S2). A unique characteristic of *OsS40* genes is that the majority of them have no introns, except *OsS40–8* and *OsS40–10* with only one or two introns (Additional file 2: Figure S1). It is proposed that genes rapidly regulated during stress contain fewer introns, since many introns may serve as negative feedback time delay loops or directly extend the length of the pre-mRNA, leading to transcription elongation [61–63]. Low intron density has also been observed in *Arabidopsis* S40 genes and reported in other stress-responsive gene families, such as the late embryogenesis abundant (LEA) gene family [64] and the trehalose-6-phosphate synthase gene family [65]. Although amino acid sequences of the rice S40 proteins do not show high similarity to HvS40 as well as AtS40–3 (Additional file 2: Figure S3), motif composition analysis provides a hint that the function of OsS40–2 may be similar to HvS40, while OsS40–14 and OsS40–16 may be similar to AtS40–3. Albeit DUF584 domain and three conserved motifs can be detected in every OsS40 protein, motif numbers and distribution alter in each of them (Additional file 2: Figure S4), resulting in disordered structure along their protein sequences, which probably contributes to structural flexibility and thereby enable the proteins to couple with different DNA, RNA or protein targets and carry versatile capability during plant senescence [66]. These results demonstrate that *OsS40* genes with few introns encode a series of DUF584 motif-possessing but unstructured proteins, which allow OsS40 proteins to function as flexible interactors to other molecules under stress conditions.

Moreover, plenty of putative TF binding sites, such as W-boxes, MYBs, MYCs and Dofs, as well as stress-responsive elements including ABRE and DRE appear in the promoter regions of rice *S40* genes (Additional file 2: Table S1), also implying that they may be regulated by distinct TFs and thus involved in developmental senescence or diverse-stress mediated signaling [67–70]. A DNA binding protein HvWHIRLY1 has been identified as a factor binding to a two-W-box element of the *HvS40* promoter in nonsenescent leaves [33]. In *Arabidopsis*, AtWHIRLY1 has been confirmed as an upstream suppressor of *AtWRKY53*, which encodes a key positive regulator of leaf senescence [71]. Therefore, HvWHIRLY1 may act as a negative regulator of *HvS40* before the onset of senescence [33]. It would be interesting to screen the rice WHIRLY-targeted promoters of *OsS40* genes by Chip-qPCR.

In general, the expression pattern of a gene reflects some connection with its function. To investigate the involvement of rice *S40* genes in natural senescence of flag leaf, the expression profiles of *OsS40* genes in different stages of flag leaf senescence were examined using semi and quantitative RT-PCR. Six of them showed increased expression at the onset or early stage of flag leaf senescence, but decreased transcript levels at late senescent leaves with less than 20% of chlorophyll, which is similar to the expression dynamic of *OSH36*, a confirmed senescence up-regulated gene in rice [40] (Fig. 2). These data indicates that a set of *OsS40* genes is probably associated with natural flag leaf senescence in a developmental age-dependent manner. According to the phylogenetic tree, OsS40–1, OsS40–2, OsS40–7 and OsS40–14 together with HvS40 and AtS40–3 belong to the same group (Fig. 1), but *OsS40–1* and *OsS40–2* were not induced during natural senescence of flag leaf. A simple and likely explanation for this is that low amino acid similarity among their proteins may result in distinct activities of *OsS40–1* and *OsS40–2* from the other members under the defined experimental conditions. Notably, it is reported that *HvS40* and *AtS40–3* transcripts accumulated to much higher levels at late senescence stage of barley primary leaves and *Arabidopsis* rosette leaves, respectively [22, 34], while the highest expression level of the six *OsS40* genes only appeared at the onset of rice flag leaf senescence. One possibility is that the tissue-dependent gene expression tendency may be inconsistent between senescent flag leaves and mature leaves, given the more important roles of flag leaf during the grain-filling stage [72, 73].

Considering that natural senescence is a complex degenerative process causing by the synergy of endogenous aging-development and environmental changes, many SAGs are also high responsive to external stresses inducing leaf senescence [2, 13, 40, 74]. *HvS40* looks like a key regulator shared by several senescence-associated pathways owing to the accumulated level of its mRNA under different conditions of senescence [19, 33, 34], while the seven *AtS40* genes induced during natural leaf senescence displayed discrete transcript profiles in respond to distinct stress-induced senescence [22]. Previously, we were also aware of the deviation of the predicted expression patterns of *OsS40* genes under salt or drought stress [24], assuming that the S40 members may play roles in crosstalk among multiple stimuli-promoted senescence pathways. To further confirm this idea, the expressions of all the 16 *OsS40* genes were tested under different stress conditions using semi and quantitative RT-PCR. The results (summarized in Table 2) reveal that three *OsS40* genes (*OsS40–4*, *OsS40–5* and *OsS40–14*) involved in the age-dependent flag leaf senescence were also up-regulated upon diverse stimuli treatments ranging from darkness to *M.oryzae* infection. It is noted that *OsS40–14* seems to be a key factor implicated in nearly all the tested stress-mediated senescent processes, except in the nitrogen deficient situation. However, the data of semi RT-PCR reflected that *OsS40–14* might be also activated during nitrogen deficiency-induced flag leaf senescence (Additional file 2: Figure S6). Thus the biological function of OsS40–14 in response to environmental stresses deserves further studies.

Similar to *AtS40–6* and *AtS40–7* [22], the other three age-associated *OsS40* genes (*OsS40–7*, *OsS40–9* and *OsS40–15*) did not show enhanced responses to various stress treatments in our test, with the exception of down-regulated *OsS40–15* in *M.oryzae*-infected rice leaves (Table 2), suggesting that they may be specifically related to developmentally controlled senescence pathways. In *Arabidopsis*, *SAG12* is identified as a natural-senescence specific marker, since its transcript level was not significantly affected by stress- or hormone-controlled senescence [13, 75]. It is predicted that the cysteine protease encoded by *SAG12* may be deleterious to the cell, resulting in its late expression during senescence [13]. Recently, it has been demonstrated that a repressor in JA-induced leaf senescence, WRKY57, is able to directly bind to the promoter of *SAG12* and disturb its transcription [76]. Therefore, we assumed *OsS40–7*, *OsS40–9* and *OsS40–15* may function downstream of the convergent senescence-associated pathways.

Additionally, we found that several *OsS40* genes, such as *OsS40–1*, *OsS40–2*, *OsS40–6* and *OsS40–12*, feature low activity or no influence during flag leaf senescence, but were dramatically induced upon external stresses or hormones treatments (Table 2), which indicates that they may be specific for stress-induced leaf senescence, albeit their transcripts presented limited similarity under distinct stress conditions. It is possible that these stress-dependent OsS40 candidates may function as rapid regulatory components to promote cell death caused by extreme environmental changes. This finding was not mentioned in the study of *Arabidopsis* S40 family, where only leaf senescence-related AtS40 members were used for further analysis. Therefore, our results extend the knowledge of potential functions of S40 proteins in the senescence-associated complex regulatory network. Gain- and loss-of function experiments may verify the importance of these OsS40 candidates in multiple aspects of rice development and senescence. However, taking account of the possible functional redundancy of these *OsS40* genes in different situations of leaf senescence, it should be noted that knockdown or knockout of these *OsS40* members may not lead to clear phenotype related to senescence, as has been displayed by deletion studies with *Arabidopsis SAG12* [77]. Nevertheless, a recent published computer-based strategy by systematically analyzing the SAG regulating network may contribute to identify key S40 genes modulating leaf senescence [78].

Due to the inclusion of the two putative nuclear localization signals (NLS) in the protein sequence, HvS40-GUS was found to mainly accumulate in the nucleus and partially in the cytoplasm [19]. Rather, most of the reported AtS40 proteins were shown to distribute in the cytoplasm, with the exception of AtS40–3, whose localization resembles that of HvS40 [22]. However, it is worth noting that the investigations on the subcellular targeting of barley HvS40 or *Arabidopsis* AtS40 proteins were conducted with onion epidermal cells and thus it is possible that the host-specific associations could have impact on the localization pattern in situ. Therefore, the OsS40 proteins, C-terminally fused to GFP, were transiently expressed in rice protoplasts. It revealed that these OsS40 members occupy discrete subcellular compartments in rice cells. OsS40–1, OsS40–13 and OsS40–14 targeted exclusively in the nucleus, while the other members either accumulated as speckles in cytoplasm or distributed in the cytoplasm (Fig. 7), suggesting that they might be regulated by various signals and may execute distinct or redundant functions in rice. In addition, as with HvS40 and AtS40–3, the putative DNA-binding property of OsS40–1 and OsS40–14 has been highlighted by the web-based protein functional families’ prediction software SVMProt [79, 80]. Usually, transcription regulators are localized in the nucleus and coupled with DNA to turn a group of target genes on or off, exemplified by OsNAP [81], OsY37 [82], OsNAC2 [83, 84], which act as positive regulators of leaf senescence. Additional experiments would be necessary to determine the DNA-binding as well as TF activity of OsS40–1 and OsS40–14.

## Conclusions

In this study, a total of 16 *S40* genes were identified in the genome of rice, which can be classified into 5 groups. Expression profiles of all these *OsS40* genes during natural senescence of flag leaf and under various senescence-promoting stress treatments uncovered that a subset of members, including *OsS40–1, OsS40–2, OsS40–12* and *OsS40–14*, were highly stress-responsive. With respect to phylogenetic relationship, transcript data and subcellular distribution, the genes *OsS40–1* and *OsS40–14* of rice are anticipated to have highest functional similarity to the *HvS40* gene of barley or the *AtS40–3* gene of *Arabidopsis*, so their functions merit further investigation. Taking together, this report provides a valuable foundation for subsequent research aimed at understanding the potential roles of S40 family in regulating plant senescence.

## Methods

### Identification and phylogenetic analysis of OsS40 proteins

To identify *S40* like genes in rice (*Oryza sativa japonica*), HvS40 protein sequence was blasted as a query sequence against the rice genome database (http://plants.ensembl.org/Oryza_sativa/Tools/Blast?db=core). ClustalW was used to perform a multiple sequence alignment of S40 proteins from rice, Arabidopsis and barley. The alignment was then analyzed for phylogenetic tress construction using neighbor joining (NJ) method by MEGA 6.0 with 1000 bootstrap replicates.

### Chromosomal location of *OsS40* genes

To map the locations of *S40* like gene transcripts in rice (*OsS40*), Chromosome Map Tool from integrated rice science database (http://viewer.shigen.info/oryzavw/maptool/Map Tool.do) was employed to visualize the chromosomal distribution. The chromosomal location information of *OsS40* genes were obtained from rice database (http://rice.plantbiology.msu.edu/).

### Characterization of gene structure and putative cis-acting elements

The exon–intron structures of *OsS40* genes were obtained by mapping the CDS to DNA sequences using the Gene Structure Display Server2.0 (http://gsds.cbi.pku.edu.cn/). CDS and genomic sequences in rice were retrieved from rice database (http://rice.plantbiology.msu.edu/). The 1-kb upstream of the transcription start site (− 1) of all identified *OsS40* transcripts were extracted as promoter to predict cis-acting elements using PlantCARE database (http://bioinformatics.psb.ugent.be/webtools/plantcare/html/).

### Conserved motif and chemical characteristics of OsS40 proteins

To discover motifs in OsS40 protein sequences, the online tool Multiple Expectation Maximization for Motif Elication (MEME) 4.11.2 (http://meme-suite.org/) was utilized to identify the conserved motifs in full-length OsS40 proteins. The optimized parameters were as follows: distribution of motifs, 0 or 1 occurrence per sequence; minimum sites, 6; maximum width 60; maximum number of motifs, 10.

To give insight into the physical and chemical characteristics of all 16 S40 proteins, 6 indexes including molecular weight, theoretical pI, number of amino acids, instability index, GRAVY and aliphatic index were calculated or predicted by Protparam program (http://web.expasy.org/protparam/).

### Plant materials and treatments

Seeds of rice cultivars, *O. sativa subsp. Japonica* and *Oryza sativa cv.* CO39, were kindly gifted from Prof. Zonghua Wang (Fujian Agriculture and Forestry University, China). After germination, rice seedlings (*O. sativa subsp. japonica*) were grown in a growth chamber in the artificial climate chamber with 12-h-light (28 °C)/12-h-dark (25 °C) photoperiod. Samples were collected at the initiation of senescence till late senescence at different time points i.e. 90DAG, 97DAG, 104DAG, 111DAG, 118DAG (DAG-Days After Germination).

To analyze nitrogen deficiency induced senescence, rice plants were grown in liquid medium containing normal N (1 ml/L), half of the normal N (1/2 ml/L) and double of the normal N (2 ml/L) conc. To analyze hormones induced senescence, detached leaves were floated in 200uM of ABA, SA, MeJA and idole acetic acid (IAA) solution. To analyze dark induced senescence, the plants were placed for 2 days in darkness.

The rice infection assay by *M. oryzae* strain Guy11 was performed. 3 to 4-week-old seedlings of rice (*Oryza sativa cv.* CO39) were inoculated by spraying fungal spores, which were suspended to a concentration of 5 × 10^4^ conidia/ml in 0.02% (*w*/*v*) Tween20 solution. Inoculated plants were kept at 20–23 °C with 80% humidity and in the dark for the first 24 h, followed by in a growth chamber at 25 °C with 80% humidity for 5 days. For RNA analysis, the infected leaves were excised and pooled at 24 h, 48 h, 72 h, 96 h and 108 h after inoculation. As a mock control, the rice seedlings were sprayed with the 0.02% (w/v) Tween20 solution only.

### Measurements of chlorophyll content and chlorophyll fluorescence

Chlorophyll content was measured using the CCM-200 plus Chlorophyll Content Meters instrument. Four leaves of each individual plant were used in the chlorophyll assay. Each leaf was placed at least for 10 points measurement. The average Fv/Fm of all rosette leaves from five individual plants was calculated. Chlorophyll fluorescence measurements were performed using Pocket PEA Chlorophyll Fluorimeter. To account for variations in photosynthetic parameters across the surface of individual plants, the data presented are the average values obtained across individual rosettes at 3 points for more than 15 min. All photosynthetic measurements were performed for four leaves of five individual plants.

### Gene expression analysis by semi quantitative RT-PCR and quantitative RT-PCR

Total RNA was extracted using the TRIzol reagent (Invitrogen, Carlsbad, CA, USA). RNA was reversely transcribed using the Transcript One-step gDNA Removal and cDNA Synthesis SuperMix. The sequences of genes related to *S40* gene family were picked from rice genome database and their coding DNA sequences were used to design gene specific primers using Premier software as well as online NCBI tools. The specific primer pairs used for semi RT-PCR are listed in Additional file 1: Table S1 and qRT-PCR in Additional file 1: Table S2. Linearity for each amplification was confirmed and the products were visualized on agarose gels stained with ethidium bromide. qRT-PCR was performed in a total volume of 20 μl, including 1× Platinium_SYBR_Green qRT-PCR SuperMix-UDG, 0.3 μM of each gene-specific primer and 10 μM of fluorescein as the passive reference dye for well-factor calibration. To calculate qRT-PCR efficiencies, three different cDNA dilutions were applied. For comparison, transcript levels of mature leaves of the treatments were normalized to control. Each data point is based on nine independent measurements including three biological replicates and three technique replicates (3 × 3).

### Construction of the plasmids

The expression vectors were constructed as follows: the coding sequences of the *OsS40* genes were cloned into the entry vector pDONR201 (Invitrogen) by using the BP-clonase, according to the manufacturer’s instructions, and subsequently cloned into the destination vector p2GWF7 (C-terminal GFP fusion-VIB, Ghent University, Belgium) by an LR reaction (Gateway recombination, Invitrogen). All of the fusion constructs were driven by the 35S promoter. The specific primer pairs used are listed in Additional file 1 Table S3.

### Protoplast isolation, transformation and confocal microscopy

The preparation of rice protoplasts was conducted based on a previously described protocol [85] with slight modifications. For isolating protoplasts, the stems and sheaths of 10–12 day-old young rice seedlings were cut into 0.5 mm strips with fresh razor blades and incubated in an enzyme solution containing 1.5% cellulase ‘Onozuka’ RS (Yakulta), 0.4% macerozyme R-10 (Yakulta) and 0.2% pectinase (Sigma). After vacuum-infiltration and enzymatic digestion, the released protoplasts were collected by filtration through 40 μm nylon meshes. Viable protoplasts were harvested by sucrose gradient centrifugation, washed once in W5 solution and then harvested by centrifugation and resuspended at a density of 2.5 × 10^6^ cells/ml in MMG solution (0.4 M mannitol, 15 mM MgCl2 and 4 mM MES at pH 5.7) prior to PEG-mediated transfection.

For transformation, 10 μg of plasmid DNA was mixed with 100 μl protoplasts (about 2.5 × 10^6^ cells/ml) and 110 μl PEG solution was added. Then, the mixture was incubated at room temperature for 5–10 min. After incubation, the mixture was diluted with 440 μL W5 solution. The solution was fully mixed by gently inverting the tubes and the protoplasts were pelleted by centrifugation at 400 g for 2 min. Transfected protoplast samples were resuspended in 200 μl W1 solution and incubated at room temperature for 10 h in darkness allowing plasmid gene expression.

All microscopic observations were performed using a Leica TCS SP8 confocal laser scanning microscope. The fluorescence of the GFP was visualized with excitation and emission wavelengths of 488 and 505–535 nm, respectively. Chloroplast auto fluorescence was visualized in a detection channel with excitation and emission wavelengths of 633 and 650–710 nm, respectively. Image processing was performed with ImageJ (http://rsb.info.nih.gov/ij/).

### Detection of GFP-fused proteins by immunoblotting

For each sample, 200 μl transformed protoplasts were collected 12 h after transfection and denatured in 20 μl protein loading buffer. SDS-PAGE and Western blot analysis were carried out using standard protocols [86]. The expression of GFP-tagged OsS40 proteins was assessed by immunoblotting using monoclonal anti-GFP antibody produced in mouse (Transgene) at a 1/3000 dilution in 5% BSA in TBS-T.

## Additional files

Additional file 1: **Table S1.** Primers used for semi qRT-PCR for expression analysis of *OsS40* genes. **Table S2.** Primers used for qRT-PCR for expression analysis of *OsS40* genes. **Table S3.** Primers used for systemic subcellular localization assays. (ZIP 244 kb)

Additional file 2: **Table S1.** Cis elements in the promoters of *S40* genes in rice, *HvS40* and *AtS40–3.* Promoter regions of 840 bp upstream of *HvS40* and 1000 bp upstream of *Ats40–3*, and rice *S40* genes were analyzed with the use of the PLACE program. W-box: Binding site for WRKY TFs; ERE: Elicitor response element; MYB: Myeloblastosis; LREs: Light regulated elements; MYC: Myelocytomatosis; ABRE: Abscisic acid responsive elements; Dof: DNA-binding with one finger; PRE: Pathogen response elements; SURE: Sulfur response elements; DRE/CRT: Dehydration response elements/C-repeat; LTR: Low temperature response; ARF: Auxin response factor; DPBFCOREDCDC3: BZIP TFs binding core sequence; G-box plus G: TF OsIRO2-binding core sequence. **Table S2.** Characteristics of rice S40 proteins. Characteristics of rice S40 proteins including theoretical isoionic point (PI), molecular weight (MW), Number of amino acids, instability index, aliphatic index and GRAVY (Grand Average of Hydropathy) predicted by ProtParam tool (http://web.expasy.org/protparam/). **Figure S1.** Exon-intron structures of *S40* genes in rice genome. Yellow color shows CDS (exon), Blue color shows UTR (untranslated regions) while normal line represents introns. **Figure S2.** Distribution of *OsS40* genes on rice chromosomes. Chromosome Map Tool was used to located genes on chromosome. **Figure S3.** Amino acid sequences of the four *Arabidopsis*, two rice and one barley protein of group I compared to the sequence of the barley HvS40 protein. The conserved DUF584 domain sequence was highlighted in black and 100% identical residues in grey. **Figure S4.** Conserved motifs in HvS40, AtS40–3 and OsS40 proteins. **a** Motif structures for the proteins were determined using MEME search tool. Grey lines represent the non-conserved sequence. Each motif is indicated by a coloered box numbered at the bottom. **b** Moti logo obtained by MEME program. The overall height of each stack represents the degree of conservation at each position, while the height of letters within each stack indicates the relative frequency of amino acids. The motifs, numbered 1–10, were displayed in different colored boxes. **Figure S5.** Semi qRT-PCR expression analysis of the sixteen *OsS40* genes at different growth stages of flag leaves, labeled as 90DAG, 97DAG, 104DAG, 111DAG) and 118DAG. DAG (Days After Germination). **Figure S6.** Semi qRT-PCR expression analysis of the sixteen *OsS40* genes at different nitrogen concentrations. Genes marked with * indicate the differentially expressed genes that were further analyzed by quantitative real-time PCR. **Figure S7.** Semi qRT-PCR expression analysis of the sixteen *OsS40* genes during dark induced leaf senescence. Detached leaves from 4-week-old rice seedlings were incubated in the deionized water in darkness for 2 days (D). As a control, the detached leaves were incubated with water at the same time in a light/dark regime (L). Genes marked with * indicate the four differentially expressed genes that were further analyzed by Real-Time PCR. **Figure S8.** Detached leaves of four weeks old rice plants were treated with different concentrations (50uM, 100uM, 200uM) of ABA, SA, MeJa and IAA. Treatment with water was used as a control. The effect of the treatment was shown by the yellowing of leaves, which initiated after 48 h of treatment with 200 μM concentration of these hormones. **Figure S9.** Semi qRT-PCR expression analysis of the sixteen *OsS40* genes in respond to ABA, SA, MeJA or IAA treatment. Treatment with water was used as a control. Among them, eight genes showed altered expression at different time of treatment. Genes marked with * indicate the eight differentially expressed genes that were further analyzed by quantitative real-time PCR. **Figure S10.** Semi qRT-PCR expression analysis of the sixteen *OsS40* genes in responds to *M. oryzae* infection. As a control the rice seedlings were sprayed with the 0.02% (*w*/*v*) Tween 20 solution only (Mock). After inoculation, the leaves were collected at 24 hpi, 48 hpi, 72 hpi, 96 hpi and 108hpi for RNA extraction. Genes marked with * indicate the ten differentially expressed genes that were further analyzed by quantitative real-time PCR. hpi, hours post-inoculation. **Figure S11.** Immunoblot analysis of C-terminal GFP-tagged OsS40 memebers transiently expressed in rice protoplasts. The corresponding GFP-tagged OsS40 proteins with expected molecular sizes are pointed out with an arrow. The actin protein and Ponceau S staining were used to check the loading level. (ZIP 14091 kb)

## Abbreviations

ABA: Abscisic acid
Chr: Chromosome
GFP: Green fluorescent protein
IAA: Idole acetic acid
MeJa: Methyl jasmonate
N: Nitrogen
qRT-PCR: Quantitative real-time polymerase chain reaction
SA: Salicylic acid
SAGs: Senescence associated genes
SAM: Shoot apical meristem
SDS-PAGE: Sodium dodecyl sulfate polyacrylamide gel electrophoresis
TFs: Transcription factors
