# Supplementary material for: Characterization of S40-like proteins and their roles in response to environmental cues and leaf senescence in rice
Source: BMC Plant Biol. 2019 May 2;19:174. doi: 10.1186/s12870-019-1767-1 (PMC6498481; doi:10.1186/s12870-019-1767-1)
Supplement: Supplementary file 1 — Table S1. Primers used for semi qRT-PCR for expression analysis of OsS40 genes. Table S2. Primers used for qRT-PCR for expression analysis of OsS40 genes. Table S3. Primers used for systemic subcellular localization assays. (ZIP 244 kb) [file 12870_2019_1767_MOESM1_ESM.zip › Additional file 1 Table S1.docx]

**Additional file 1: Table S1**

**Table S1**. Primers used for semi qRT-PCR for expression analysis of *OsS40* genes.

| **Gene Name** | **Forward primer** | **Reverse primer** |
| --- | --- | --- |
| *OsS40-1* | CGTCGTCGTTGTCGTCTCC | CTCGATGAATCCGGTCATCTG |
| *OsS40-2* | ATGGAGGAGTTCCAAGAAGCC | TCTCGAGGAAGCCGGTCAT |
| *OsS40-3* | CCAGTCGGCTCCCGTGAAG | TCGTCGTCGTCGTCGTCGTAT |
| *OsS40-4* | AATCCAAAGCATCCCATCGC | TCTCTAATTCCCAGAGACCAATC |
| *OsS40-5* | ACCAATTCCTCGCCGTCC | TCGTCGTCATCGTCCTCCTC |
| *OsS40-6* | GCCTCCTCAACCAATTCCTC | TCGTCGTCATCGTCCTCCTC |
| *OsS40-7* | TGTACGAGTCCGACGTGCTGT | AGTTGCGCAGGTGGCTGAG |
| *OsS40-8* | GCGGGAAGGGGAAGCGAG | GGTCTTCTCCCAGATGGCGTTG |
| *OsS40-9* | CCTCGATTGATCCGTGCCTT | GAAGTATCCACCATGAGGTCACA |
| *OsS40-10* | TGGGCACTTGGGCATTACAC | TCGTGCCGAAGGGGAGGGCG |
| *OsS40-11* | CCGACGACGAGGGGAGCAAG | TGGAGCGTCCTCCCGGCG |
| *OsS40-12* | CGAAACCGACCTCTGCCAATG | TCAGTCGAGGAAGCCGGTGC |
| *OsS40-13* | GCACGCCTACCGGATGTTCG | CCACCTTGCCGTACTCGTAGTCG |
| *OsS40-14* | TGGAGATACGGTGGTGCCAG | TCATCCGCAGCACCGAGTTG |
| *OsS40-15* | TAAACCGGGGAGACAAGCCA | CCCAACCACCACCCAATCAA |
| *OsS40-16* | GACGAGGGAGAAGTGTGGGAAG | GCGTTCATCCGCTCCATCTT |
