# Supplementary material for: Characterization of S40-like proteins and their roles in response to environmental cues and leaf senescence in rice
Source: BMC Plant Biol. 2019 May 2;19:174. doi: 10.1186/s12870-019-1767-1 (PMC6498481; doi:10.1186/s12870-019-1767-1)
Supplement: Supplementary file 1 — Table S1. Primers used for semi qRT-PCR for expression analysis of OsS40 genes. Table S2. Primers used for qRT-PCR for expression analysis of OsS40 genes. Table S3. Primers used for systemic subcellular localization assays. (ZIP 244 kb) [file 12870_2019_1767_MOESM1_ESM.zip › Additional file 1 Table S2.docx]

**Additional file 1: Table S2**

**Table S2.** Primers used for qRT-PCR for expression analysis of *OsS40* genes.

| **Gene Name** | **Forward primer** | **Reverse primer** |
| --- | --- | --- |
| *OsS40-1* | ACGACGCTGTGAAGAAGAAGATG | AGCCTCCTCCGATCAACAATG |
| *OsS40-2* | GAGGATCAGCCACGGGAGC | TCCCCACGCACACCGAGAA |
| *OsS40-3* | CCCAGGGTGAGGTTCCAC | GTCGTCGTATCCGTCGCC |
| *OsS40-4* | ACGAGGACGACGAGATGGTG | CAGAGGTCGAGGAATCCGGT |
| *OsS40-5* | AACCAATTCCTCGCCGTCC | GTCGTCATCGTCCTCCTCGTC |
| *OsS40-6* | TTGTCGCCAACCCAGTCC | CGTCCCACTTGAACATCTCCT |
| *OsS40-7* | GCTGTGGCCGGATCACC | GATCTGCTGCTGGCCCG |
| *OsS40-8* | GTCGAAGGGCGGGAAGGGGA | CTCCCAGATGGCGTTGCGG |
| *OsS40-9* | TAGGCGGTTATCGGTGGAGG | CGAGAAGGTGGTCATCGGTG |
| *OsS40-10* | ATGGGCACTTGGGCATTACACC | TCCAGGTCTTGTGCTGCATCTG |
| *OsS40-11* | CGACGACGAGGGGAGCAAGA | TCCGAGGGTATCCTCACGGG |
| *OsS40-12* | GACGTGCTCTGGCCTGCAT | CGCCTCCGACGGTATCCTC |
| *OsS40-13* | CGAGTTCGACGAGTCGGA | CCTGTACTCGACCCCAAGAA |
| *OsS40-14* | GTGGAGGAGCTCGACGAGTT | GTGTCTCGTATGGCTGCACC |
| *OsS40-15* | GTTCCAGTACCACCACCACC | CGTACCAGACATCAGCCTCC |
| *OsS40-16* | TCACGATCAGAGCAACGAAGCG | TCCTCGTGCTGCCTGTTATTCC |
| *Actin* | TCCATCTTGGCATCTCTCAG | GTACCCTCATCAGGCATCTG |
| *OSH36* | GCACGGAGGCGAACGA | TTGAGCGGTAGCACCCATT |
| *OsNAP* | CAAGAAGCCGAACGGTTC | GTTAGAGTGGAGCAGCAT |
| *SGR* | AGGGGTGGTACAACAAGCTG | GCTCCTTGCGGAAGATGTAG |
| *NYC1* | CATGCAACACCAACAAAAGG | GACCATTCCAGGAGAAGCAG |
| *NYC3* | TGTCGTTGCCATGTGAAGAT | TTGGTCACGCCACAAATCTA |
| *Os157* | ACCCTAAAGTAAATGAAGTC | CCTGCTCTTGTCTTGTTA |
| *OsWRKY45* | CGGGTAAAACGATCGAAAGA | TTTCGAAAGCGGAAGAACAG |
| *OsNAC4* | TCCTGCCACCATTCTGAGATG | TTGCAGAATCATGCTTGCCAG |
