# Supplementary material for: Characterization of S40-like proteins and their roles in response to environmental cues and leaf senescence in rice
Source: BMC Plant Biol. 2019 May 2;19:174. doi: 10.1186/s12870-019-1767-1 (PMC6498481; doi:10.1186/s12870-019-1767-1)
Supplement: Supplementary file 1 — Table S1. Primers used for semi qRT-PCR for expression analysis of OsS40 genes. Table S2. Primers used for qRT-PCR for expression analysis of OsS40 genes. Table S3. Primers used for systemic subcellular localization assays. (ZIP 244 kb) [file 12870_2019_1767_MOESM1_ESM.zip › Additional file 1 Table S3.docx]

**Additional file 1: Table S3**

**Table S3**. Primers used for systemic subcellular localization assays.

| OsS40-1 attB1 | AAAAAGCAGGCTTCACCATGGAGGAATTCCAAGAGG |
| --- | --- |
| OsS40-1 attB2 | AGAAAGCTGGGTCTTTCTCGATGAATCCGGTC |
| OsS40-3 attB1 | AAAAAGCAGGCTTCACCATGGACTCCGCCGCCC |
| OsS40-3 attB2 | AGAAAGCTGGGTCGTCAAGAAATCCGGTTTGCC |
| OsS40-6 attB1 | AAAAAGCAGGCTTCACCATGGACGAGTACAGGCC |
| OsS40-6 attB2 | AGAAAGCTGGGTCGTCGAGGAAACCGGTT |
| OsS40-7 attB1 | AAAAAGCAGGCTTCACCATGGCGACGATGGGAGAG |
| OsS40-7 attB2 | AGAAAGCTGGGTCTCCTTCAATGAACCCGGTC |
| OsS40-9 attB1 | AAAAAGCAGGCTTCACCATGGACCGCTCTCGCCA |
| OsS40-9 attB2 | AGAAAGCTGGGTCATCGAGGAATCCTGTCTTCCG |
| OsS40-13 attB1 | AAAAAGCAGGCTTCACCATGGCTGGGAGCGCGA |
| OsS40-13 attB2 | AGAAAGCTGGGTCGTCCTCGAAGCCGGTCTTC |
| OsS40-14 attB1 | AAAAAGCAGGCTTCACCATGGCGATGGTGGTGGA |
| OsS40-14 attB2 | AGAAAGCTGGGTCTCCTTCGATGAACCCGG |
| OsS40-15 attB1 | AAAAAGCAGGCTTCACCATGGCGAAGGCGCGGAAG |
| OsS40-15 attB2 | AGAAAGCTGGGTCGCCGTCGAAGCCGGTTCG |
| OsS40-16 attB1 | AAAAAGCAGGCTTCACCATGGCGGGAGTGGTACGTC |
| OsS40-16 attB2 | AGAAAGCTGGGTCCTGCTCCGATGAGAAGGCG |
| attB1-adapter | GGGGACAAGTTTGTACAAAAAAGCAGGCT |
| attB2-adapter | GGGGACCACTTTGTACAAGAAAGCTGGGT |
