# Supplementary figures and images for: Characterization of S40-like proteins and their roles in response to environmental cues and leaf senescence in rice
Source: BMC Plant Biol. 2019 May 2;19:174. doi: 10.1186/s12870-019-1767-1 (PMC6498481; doi:10.1186/s12870-019-1767-1)

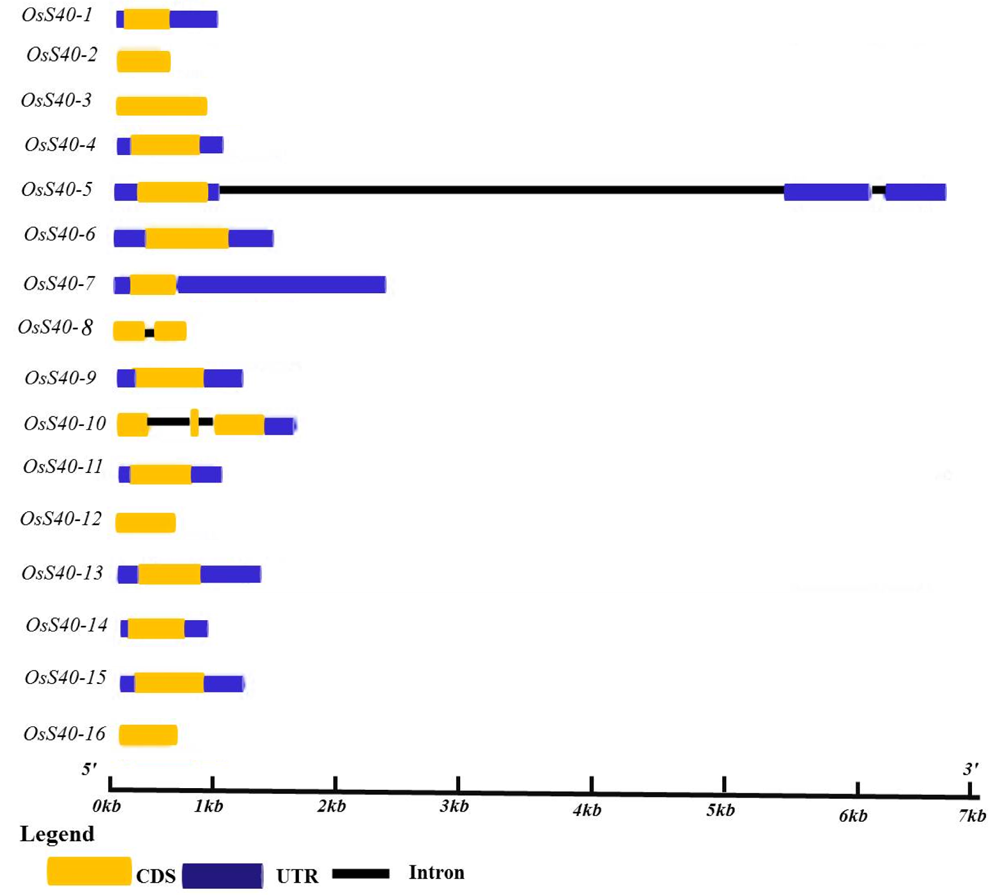

Supplement: Supplementary file 2 — Table S1. Cis elements in the promoters of S40 genes in rice, HvS40 and AtS40–3. Promoter regions of 840 bp upstream of HvS40 and 1000 bp upstream of Ats40–3, and rice S40 genes were analyzed with the use of the PLACE program. W-box: Binding site for WRKY TFs; ERE: Elicitor response element; MYB: Myeloblastosis; LREs: Light regulated elements; MYC: Myelocytomatosis; ABRE: Abscisic acid responsive elements; Dof: DNA-binding with one finger; PRE: Pathogen response elements; SURE: Sulfur response elements; DRE/CRT: Dehydration response elements/C-repeat; LTR: Low temperature response; ARF: Auxin response factor; DPBFCOREDCDC3: BZIP TFs binding core sequence; G-box plus G: TF OsIRO2-binding core sequence. Table S2. Characteristics of rice S40 proteins. Characteristics of rice S40 proteins including theoretical isoionic point (PI), molecular weight (MW), Number of amino acids, instability index, aliphatic index and GRAVY (Grand Average of Hydropathy) predicted by ProtParam tool (http://web.expasy.org/protparam/). Figure S1. Exon-intron structures of S40 genes in rice genome. Yellow color shows CDS (exon), Blue color shows UTR (untranslated regions) while normal line represents introns. Figure S2. Distribution of OsS40 genes on rice chromosomes. Chromosome Map Tool was used to located genes on chromosome. Figure S3. Amino acid sequences of the four Arabidopsis, two rice and one barley protein of group I compared to the sequence of the barley HvS40 protein. The conserved DUF584 domain sequence was highlighted in black and 100% identical residues in grey. Figure S4. Conserved motifs in HvS40, AtS40–3 and OsS40 proteins. a Motif structures for the proteins were determined using MEME search tool. Grey lines represent the non-conserved sequence. Each motif is indicated by a coloered box numbered at the bottom. b Moti logo obtained by MEME program. The overall height of each stack represents the degree of conservation at each position, while the height of letters within each st [file 12870_2019_1767_MOESM2_ESM.zip › Additional file 2 Figure S1.tif]

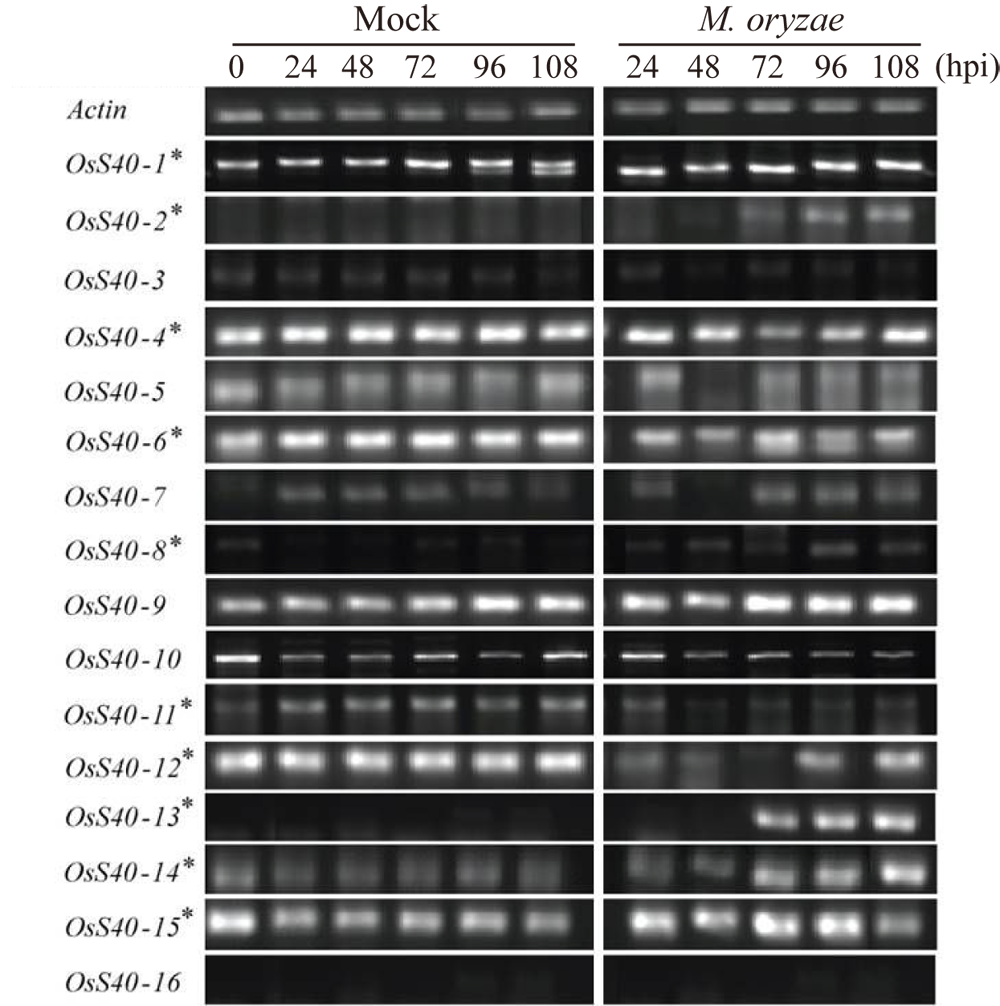

Supplement: Supplementary file 2 — Table S1. Cis elements in the promoters of S40 genes in rice, HvS40 and AtS40–3. Promoter regions of 840 bp upstream of HvS40 and 1000 bp upstream of Ats40–3, and rice S40 genes were analyzed with the use of the PLACE program. W-box: Binding site for WRKY TFs; ERE: Elicitor response element; MYB: Myeloblastosis; LREs: Light regulated elements; MYC: Myelocytomatosis; ABRE: Abscisic acid responsive elements; Dof: DNA-binding with one finger; PRE: Pathogen response elements; SURE: Sulfur response elements; DRE/CRT: Dehydration response elements/C-repeat; LTR: Low temperature response; ARF: Auxin response factor; DPBFCOREDCDC3: BZIP TFs binding core sequence; G-box plus G: TF OsIRO2-binding core sequence. Table S2. Characteristics of rice S40 proteins. Characteristics of rice S40 proteins including theoretical isoionic point (PI), molecular weight (MW), Number of amino acids, instability index, aliphatic index and GRAVY (Grand Average of Hydropathy) predicted by ProtParam tool (http://web.expasy.org/protparam/). Figure S1. Exon-intron structures of S40 genes in rice genome. Yellow color shows CDS (exon), Blue color shows UTR (untranslated regions) while normal line represents introns. Figure S2. Distribution of OsS40 genes on rice chromosomes. Chromosome Map Tool was used to located genes on chromosome. Figure S3. Amino acid sequences of the four Arabidopsis, two rice and one barley protein of group I compared to the sequence of the barley HvS40 protein. The conserved DUF584 domain sequence was highlighted in black and 100% identical residues in grey. Figure S4. Conserved motifs in HvS40, AtS40–3 and OsS40 proteins. a Motif structures for the proteins were determined using MEME search tool. Grey lines represent the non-conserved sequence. Each motif is indicated by a coloered box numbered at the bottom. b Moti logo obtained by MEME program. The overall height of each stack represents the degree of conservation at each position, while the height of letters within each st [file 12870_2019_1767_MOESM2_ESM.zip › Additional file 2 Figure S10.tif]

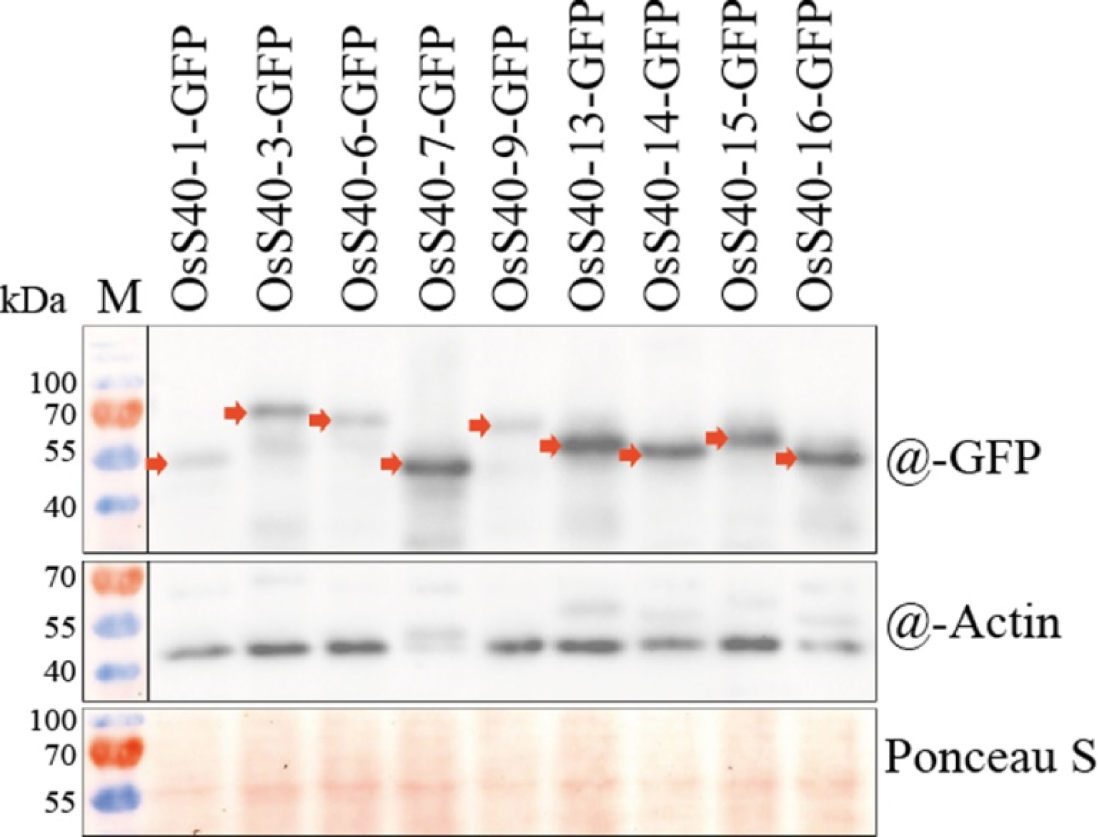

Supplement: Supplementary file 2 — Table S1. Cis elements in the promoters of S40 genes in rice, HvS40 and AtS40–3. Promoter regions of 840 bp upstream of HvS40 and 1000 bp upstream of Ats40–3, and rice S40 genes were analyzed with the use of the PLACE program. W-box: Binding site for WRKY TFs; ERE: Elicitor response element; MYB: Myeloblastosis; LREs: Light regulated elements; MYC: Myelocytomatosis; ABRE: Abscisic acid responsive elements; Dof: DNA-binding with one finger; PRE: Pathogen response elements; SURE: Sulfur response elements; DRE/CRT: Dehydration response elements/C-repeat; LTR: Low temperature response; ARF: Auxin response factor; DPBFCOREDCDC3: BZIP TFs binding core sequence; G-box plus G: TF OsIRO2-binding core sequence. Table S2. Characteristics of rice S40 proteins. Characteristics of rice S40 proteins including theoretical isoionic point (PI), molecular weight (MW), Number of amino acids, instability index, aliphatic index and GRAVY (Grand Average of Hydropathy) predicted by ProtParam tool (http://web.expasy.org/protparam/). Figure S1. Exon-intron structures of S40 genes in rice genome. Yellow color shows CDS (exon), Blue color shows UTR (untranslated regions) while normal line represents introns. Figure S2. Distribution of OsS40 genes on rice chromosomes. Chromosome Map Tool was used to located genes on chromosome. Figure S3. Amino acid sequences of the four Arabidopsis, two rice and one barley protein of group I compared to the sequence of the barley HvS40 protein. The conserved DUF584 domain sequence was highlighted in black and 100% identical residues in grey. Figure S4. Conserved motifs in HvS40, AtS40–3 and OsS40 proteins. a Motif structures for the proteins were determined using MEME search tool. Grey lines represent the non-conserved sequence. Each motif is indicated by a coloered box numbered at the bottom. b Moti logo obtained by MEME program. The overall height of each stack represents the degree of conservation at each position, while the height of letters within each st [file 12870_2019_1767_MOESM2_ESM.zip › Additional file 2 Figure S11.tif]

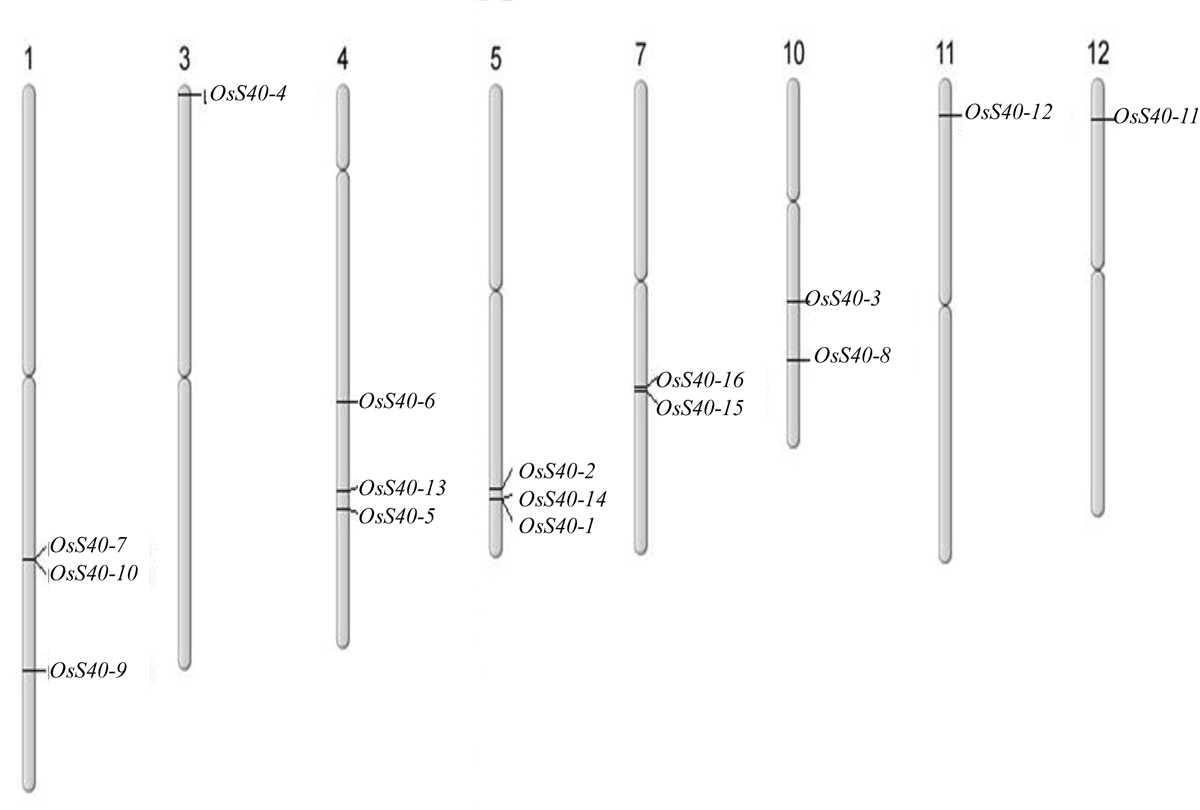

Supplement: Supplementary file 2 — Table S1. Cis elements in the promoters of S40 genes in rice, HvS40 and AtS40–3. Promoter regions of 840 bp upstream of HvS40 and 1000 bp upstream of Ats40–3, and rice S40 genes were analyzed with the use of the PLACE program. W-box: Binding site for WRKY TFs; ERE: Elicitor response element; MYB: Myeloblastosis; LREs: Light regulated elements; MYC: Myelocytomatosis; ABRE: Abscisic acid responsive elements; Dof: DNA-binding with one finger; PRE: Pathogen response elements; SURE: Sulfur response elements; DRE/CRT: Dehydration response elements/C-repeat; LTR: Low temperature response; ARF: Auxin response factor; DPBFCOREDCDC3: BZIP TFs binding core sequence; G-box plus G: TF OsIRO2-binding core sequence. Table S2. Characteristics of rice S40 proteins. Characteristics of rice S40 proteins including theoretical isoionic point (PI), molecular weight (MW), Number of amino acids, instability index, aliphatic index and GRAVY (Grand Average of Hydropathy) predicted by ProtParam tool (http://web.expasy.org/protparam/). Figure S1. Exon-intron structures of S40 genes in rice genome. Yellow color shows CDS (exon), Blue color shows UTR (untranslated regions) while normal line represents introns. Figure S2. Distribution of OsS40 genes on rice chromosomes. Chromosome Map Tool was used to located genes on chromosome. Figure S3. Amino acid sequences of the four Arabidopsis, two rice and one barley protein of group I compared to the sequence of the barley HvS40 protein. The conserved DUF584 domain sequence was highlighted in black and 100% identical residues in grey. Figure S4. Conserved motifs in HvS40, AtS40–3 and OsS40 proteins. a Motif structures for the proteins were determined using MEME search tool. Grey lines represent the non-conserved sequence. Each motif is indicated by a coloered box numbered at the bottom. b Moti logo obtained by MEME program. The overall height of each stack represents the degree of conservation at each position, while the height of letters within each st [file 12870_2019_1767_MOESM2_ESM.zip › Additional file 2 Figure S2.tif]

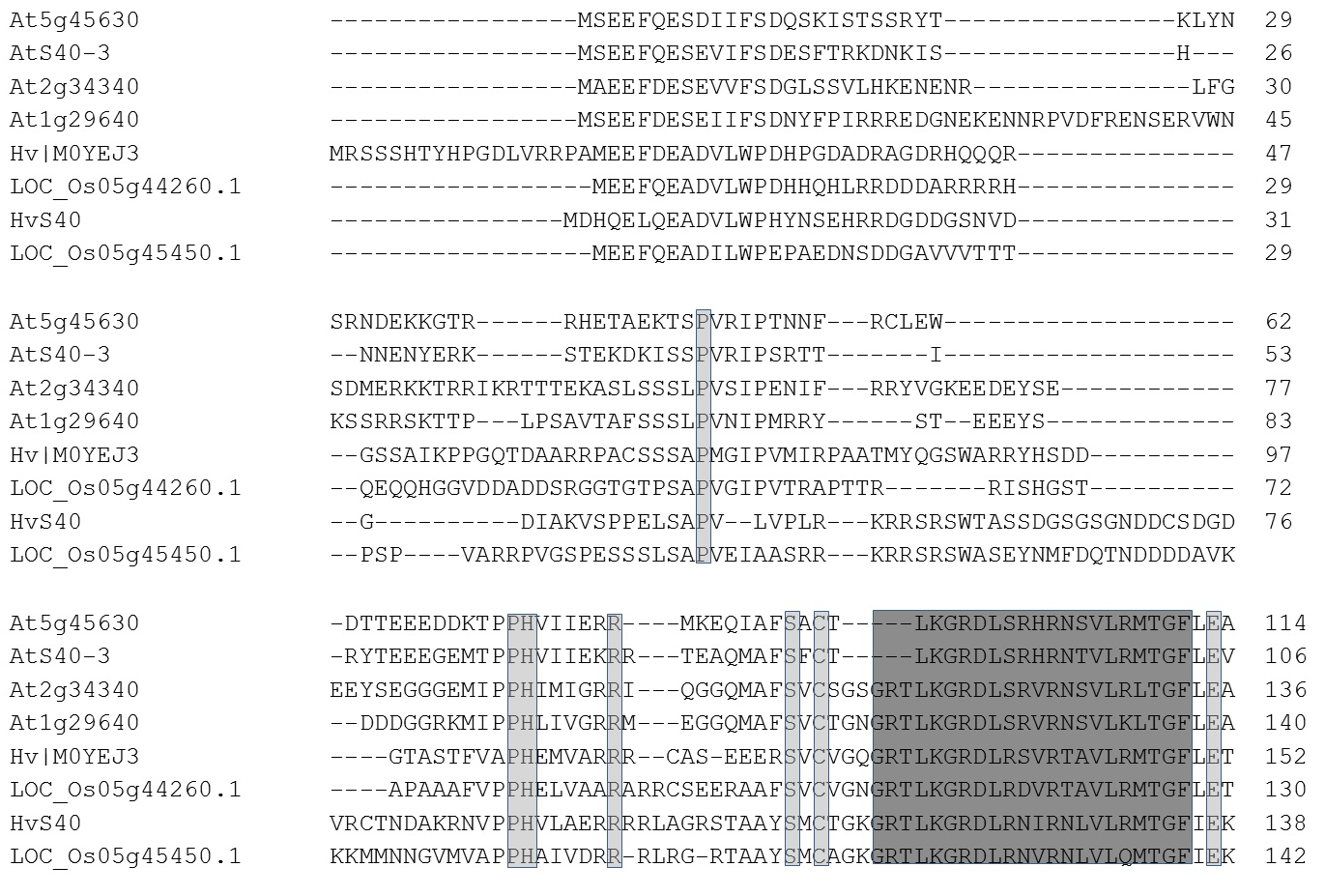

Supplement: Supplementary file 2 — Table S1. Cis elements in the promoters of S40 genes in rice, HvS40 and AtS40–3. Promoter regions of 840 bp upstream of HvS40 and 1000 bp upstream of Ats40–3, and rice S40 genes were analyzed with the use of the PLACE program. W-box: Binding site for WRKY TFs; ERE: Elicitor response element; MYB: Myeloblastosis; LREs: Light regulated elements; MYC: Myelocytomatosis; ABRE: Abscisic acid responsive elements; Dof: DNA-binding with one finger; PRE: Pathogen response elements; SURE: Sulfur response elements; DRE/CRT: Dehydration response elements/C-repeat; LTR: Low temperature response; ARF: Auxin response factor; DPBFCOREDCDC3: BZIP TFs binding core sequence; G-box plus G: TF OsIRO2-binding core sequence. Table S2. Characteristics of rice S40 proteins. Characteristics of rice S40 proteins including theoretical isoionic point (PI), molecular weight (MW), Number of amino acids, instability index, aliphatic index and GRAVY (Grand Average of Hydropathy) predicted by ProtParam tool (http://web.expasy.org/protparam/). Figure S1. Exon-intron structures of S40 genes in rice genome. Yellow color shows CDS (exon), Blue color shows UTR (untranslated regions) while normal line represents introns. Figure S2. Distribution of OsS40 genes on rice chromosomes. Chromosome Map Tool was used to located genes on chromosome. Figure S3. Amino acid sequences of the four Arabidopsis, two rice and one barley protein of group I compared to the sequence of the barley HvS40 protein. The conserved DUF584 domain sequence was highlighted in black and 100% identical residues in grey. Figure S4. Conserved motifs in HvS40, AtS40–3 and OsS40 proteins. a Motif structures for the proteins were determined using MEME search tool. Grey lines represent the non-conserved sequence. Each motif is indicated by a coloered box numbered at the bottom. b Moti logo obtained by MEME program. The overall height of each stack represents the degree of conservation at each position, while the height of letters within each st [file 12870_2019_1767_MOESM2_ESM.zip › Additional file 2 Figure S3.tif]

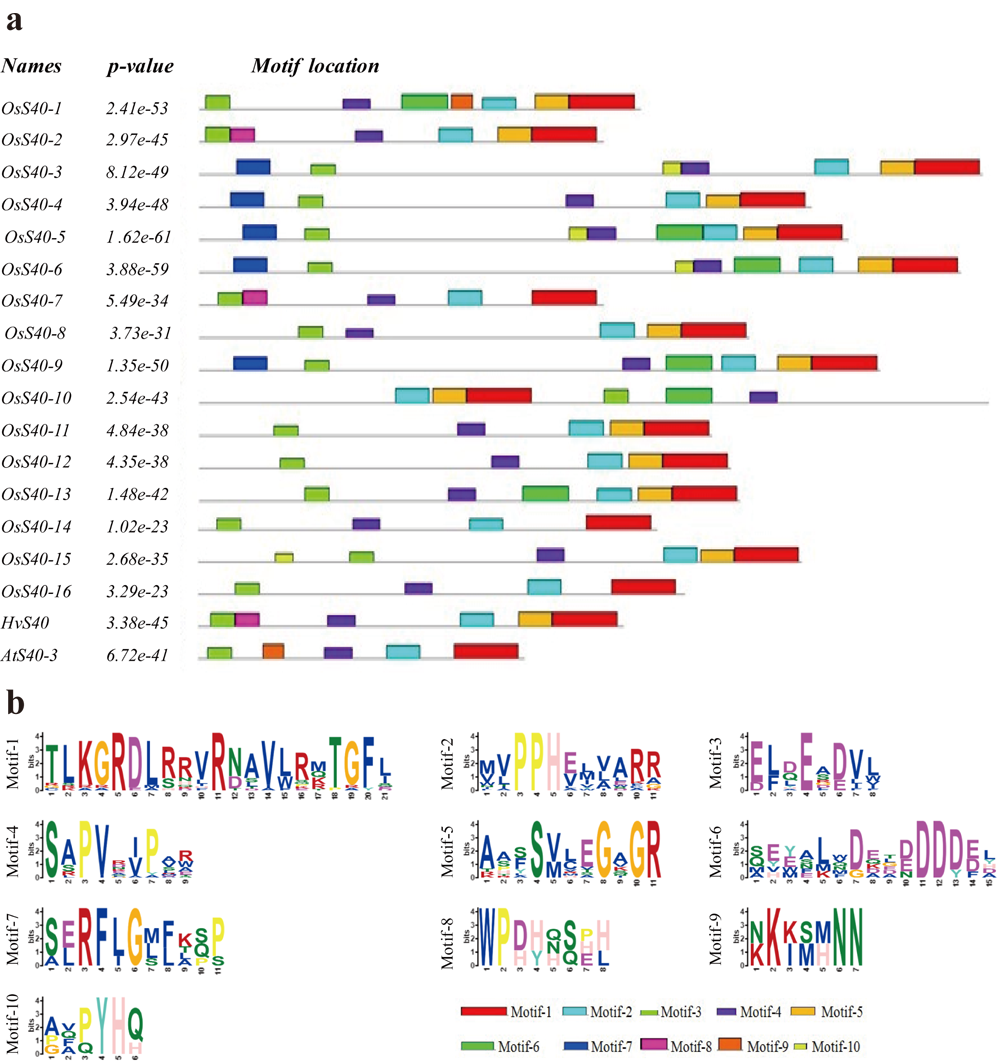

Supplement: Supplementary file 2 — Table S1. Cis elements in the promoters of S40 genes in rice, HvS40 and AtS40–3. Promoter regions of 840 bp upstream of HvS40 and 1000 bp upstream of Ats40–3, and rice S40 genes were analyzed with the use of the PLACE program. W-box: Binding site for WRKY TFs; ERE: Elicitor response element; MYB: Myeloblastosis; LREs: Light regulated elements; MYC: Myelocytomatosis; ABRE: Abscisic acid responsive elements; Dof: DNA-binding with one finger; PRE: Pathogen response elements; SURE: Sulfur response elements; DRE/CRT: Dehydration response elements/C-repeat; LTR: Low temperature response; ARF: Auxin response factor; DPBFCOREDCDC3: BZIP TFs binding core sequence; G-box plus G: TF OsIRO2-binding core sequence. Table S2. Characteristics of rice S40 proteins. Characteristics of rice S40 proteins including theoretical isoionic point (PI), molecular weight (MW), Number of amino acids, instability index, aliphatic index and GRAVY (Grand Average of Hydropathy) predicted by ProtParam tool (http://web.expasy.org/protparam/). Figure S1. Exon-intron structures of S40 genes in rice genome. Yellow color shows CDS (exon), Blue color shows UTR (untranslated regions) while normal line represents introns. Figure S2. Distribution of OsS40 genes on rice chromosomes. Chromosome Map Tool was used to located genes on chromosome. Figure S3. Amino acid sequences of the four Arabidopsis, two rice and one barley protein of group I compared to the sequence of the barley HvS40 protein. The conserved DUF584 domain sequence was highlighted in black and 100% identical residues in grey. Figure S4. Conserved motifs in HvS40, AtS40–3 and OsS40 proteins. a Motif structures for the proteins were determined using MEME search tool. Grey lines represent the non-conserved sequence. Each motif is indicated by a coloered box numbered at the bottom. b Moti logo obtained by MEME program. The overall height of each stack represents the degree of conservation at each position, while the height of letters within each st [file 12870_2019_1767_MOESM2_ESM.zip › Additional file 2 Figure S4.tif]

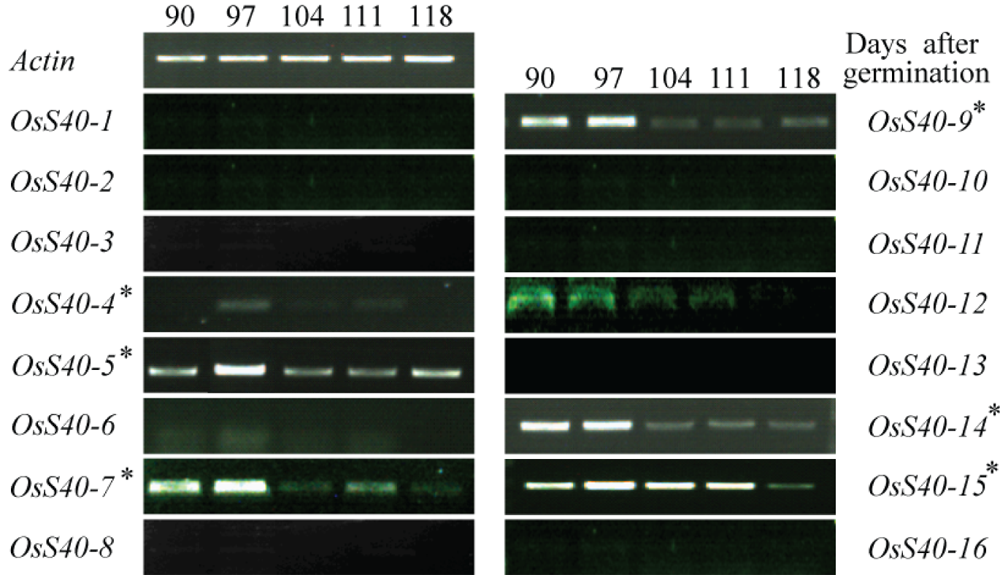

Supplement: Supplementary file 2 — Table S1. Cis elements in the promoters of S40 genes in rice, HvS40 and AtS40–3. Promoter regions of 840 bp upstream of HvS40 and 1000 bp upstream of Ats40–3, and rice S40 genes were analyzed with the use of the PLACE program. W-box: Binding site for WRKY TFs; ERE: Elicitor response element; MYB: Myeloblastosis; LREs: Light regulated elements; MYC: Myelocytomatosis; ABRE: Abscisic acid responsive elements; Dof: DNA-binding with one finger; PRE: Pathogen response elements; SURE: Sulfur response elements; DRE/CRT: Dehydration response elements/C-repeat; LTR: Low temperature response; ARF: Auxin response factor; DPBFCOREDCDC3: BZIP TFs binding core sequence; G-box plus G: TF OsIRO2-binding core sequence. Table S2. Characteristics of rice S40 proteins. Characteristics of rice S40 proteins including theoretical isoionic point (PI), molecular weight (MW), Number of amino acids, instability index, aliphatic index and GRAVY (Grand Average of Hydropathy) predicted by ProtParam tool (http://web.expasy.org/protparam/). Figure S1. Exon-intron structures of S40 genes in rice genome. Yellow color shows CDS (exon), Blue color shows UTR (untranslated regions) while normal line represents introns. Figure S2. Distribution of OsS40 genes on rice chromosomes. Chromosome Map Tool was used to located genes on chromosome. Figure S3. Amino acid sequences of the four Arabidopsis, two rice and one barley protein of group I compared to the sequence of the barley HvS40 protein. The conserved DUF584 domain sequence was highlighted in black and 100% identical residues in grey. Figure S4. Conserved motifs in HvS40, AtS40–3 and OsS40 proteins. a Motif structures for the proteins were determined using MEME search tool. Grey lines represent the non-conserved sequence. Each motif is indicated by a coloered box numbered at the bottom. b Moti logo obtained by MEME program. The overall height of each stack represents the degree of conservation at each position, while the height of letters within each st [file 12870_2019_1767_MOESM2_ESM.zip › Additional file 2 Figure S5.tif]

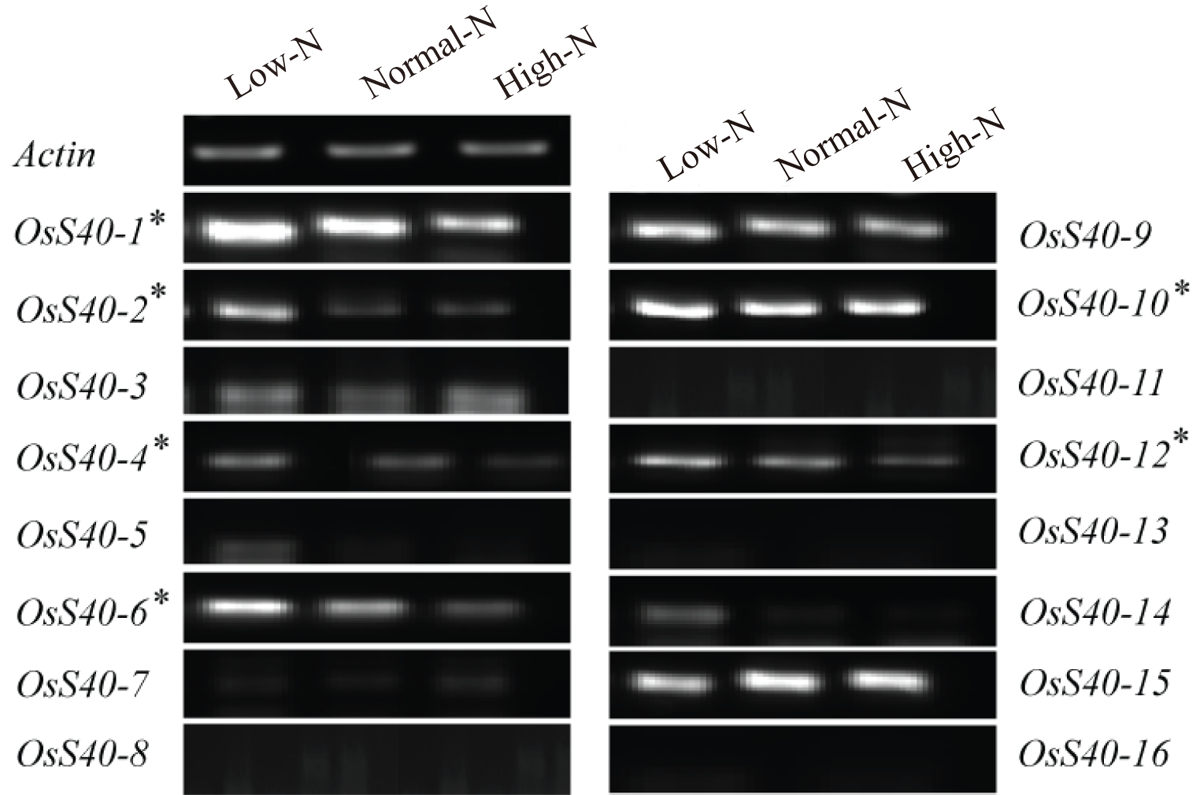

Supplement: Supplementary file 2 — Table S1. Cis elements in the promoters of S40 genes in rice, HvS40 and AtS40–3. Promoter regions of 840 bp upstream of HvS40 and 1000 bp upstream of Ats40–3, and rice S40 genes were analyzed with the use of the PLACE program. W-box: Binding site for WRKY TFs; ERE: Elicitor response element; MYB: Myeloblastosis; LREs: Light regulated elements; MYC: Myelocytomatosis; ABRE: Abscisic acid responsive elements; Dof: DNA-binding with one finger; PRE: Pathogen response elements; SURE: Sulfur response elements; DRE/CRT: Dehydration response elements/C-repeat; LTR: Low temperature response; ARF: Auxin response factor; DPBFCOREDCDC3: BZIP TFs binding core sequence; G-box plus G: TF OsIRO2-binding core sequence. Table S2. Characteristics of rice S40 proteins. Characteristics of rice S40 proteins including theoretical isoionic point (PI), molecular weight (MW), Number of amino acids, instability index, aliphatic index and GRAVY (Grand Average of Hydropathy) predicted by ProtParam tool (http://web.expasy.org/protparam/). Figure S1. Exon-intron structures of S40 genes in rice genome. Yellow color shows CDS (exon), Blue color shows UTR (untranslated regions) while normal line represents introns. Figure S2. Distribution of OsS40 genes on rice chromosomes. Chromosome Map Tool was used to located genes on chromosome. Figure S3. Amino acid sequences of the four Arabidopsis, two rice and one barley protein of group I compared to the sequence of the barley HvS40 protein. The conserved DUF584 domain sequence was highlighted in black and 100% identical residues in grey. Figure S4. Conserved motifs in HvS40, AtS40–3 and OsS40 proteins. a Motif structures for the proteins were determined using MEME search tool. Grey lines represent the non-conserved sequence. Each motif is indicated by a coloered box numbered at the bottom. b Moti logo obtained by MEME program. The overall height of each stack represents the degree of conservation at each position, while the height of letters within each st [file 12870_2019_1767_MOESM2_ESM.zip › Additional file 2 Figure S6.tif]

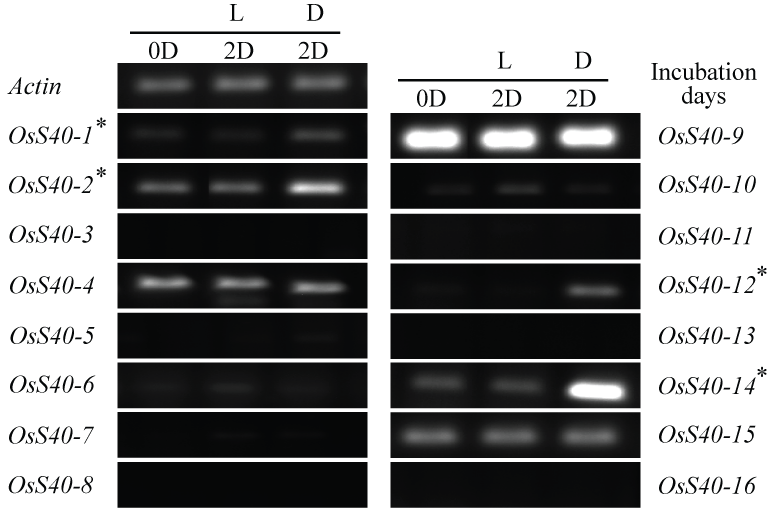

Supplement: Supplementary file 2 — Table S1. Cis elements in the promoters of S40 genes in rice, HvS40 and AtS40–3. Promoter regions of 840 bp upstream of HvS40 and 1000 bp upstream of Ats40–3, and rice S40 genes were analyzed with the use of the PLACE program. W-box: Binding site for WRKY TFs; ERE: Elicitor response element; MYB: Myeloblastosis; LREs: Light regulated elements; MYC: Myelocytomatosis; ABRE: Abscisic acid responsive elements; Dof: DNA-binding with one finger; PRE: Pathogen response elements; SURE: Sulfur response elements; DRE/CRT: Dehydration response elements/C-repeat; LTR: Low temperature response; ARF: Auxin response factor; DPBFCOREDCDC3: BZIP TFs binding core sequence; G-box plus G: TF OsIRO2-binding core sequence. Table S2. Characteristics of rice S40 proteins. Characteristics of rice S40 proteins including theoretical isoionic point (PI), molecular weight (MW), Number of amino acids, instability index, aliphatic index and GRAVY (Grand Average of Hydropathy) predicted by ProtParam tool (http://web.expasy.org/protparam/). Figure S1. Exon-intron structures of S40 genes in rice genome. Yellow color shows CDS (exon), Blue color shows UTR (untranslated regions) while normal line represents introns. Figure S2. Distribution of OsS40 genes on rice chromosomes. Chromosome Map Tool was used to located genes on chromosome. Figure S3. Amino acid sequences of the four Arabidopsis, two rice and one barley protein of group I compared to the sequence of the barley HvS40 protein. The conserved DUF584 domain sequence was highlighted in black and 100% identical residues in grey. Figure S4. Conserved motifs in HvS40, AtS40–3 and OsS40 proteins. a Motif structures for the proteins were determined using MEME search tool. Grey lines represent the non-conserved sequence. Each motif is indicated by a coloered box numbered at the bottom. b Moti logo obtained by MEME program. The overall height of each stack represents the degree of conservation at each position, while the height of letters within each st [file 12870_2019_1767_MOESM2_ESM.zip › Additional file 2 Figure S7.tif]

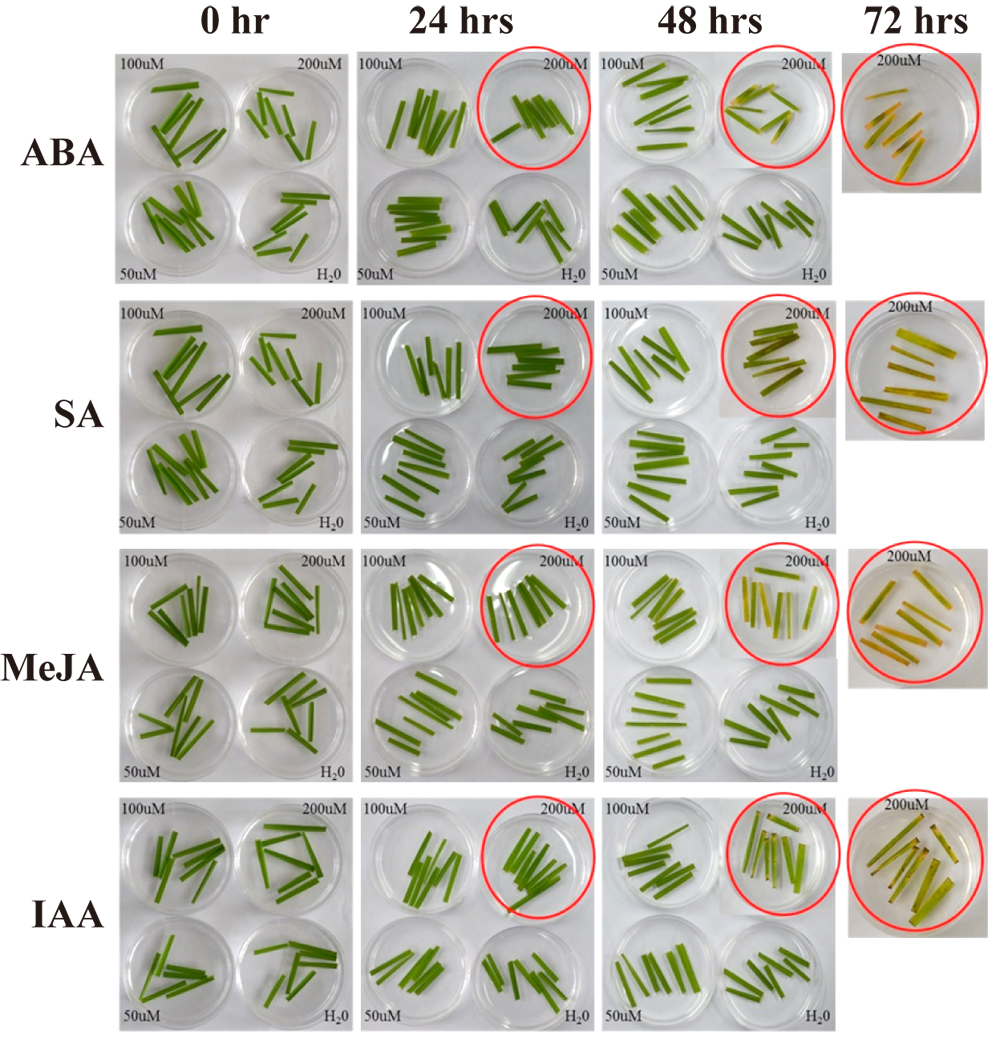

Supplement: Supplementary file 2 — Table S1. Cis elements in the promoters of S40 genes in rice, HvS40 and AtS40–3. Promoter regions of 840 bp upstream of HvS40 and 1000 bp upstream of Ats40–3, and rice S40 genes were analyzed with the use of the PLACE program. W-box: Binding site for WRKY TFs; ERE: Elicitor response element; MYB: Myeloblastosis; LREs: Light regulated elements; MYC: Myelocytomatosis; ABRE: Abscisic acid responsive elements; Dof: DNA-binding with one finger; PRE: Pathogen response elements; SURE: Sulfur response elements; DRE/CRT: Dehydration response elements/C-repeat; LTR: Low temperature response; ARF: Auxin response factor; DPBFCOREDCDC3: BZIP TFs binding core sequence; G-box plus G: TF OsIRO2-binding core sequence. Table S2. Characteristics of rice S40 proteins. Characteristics of rice S40 proteins including theoretical isoionic point (PI), molecular weight (MW), Number of amino acids, instability index, aliphatic index and GRAVY (Grand Average of Hydropathy) predicted by ProtParam tool (http://web.expasy.org/protparam/). Figure S1. Exon-intron structures of S40 genes in rice genome. Yellow color shows CDS (exon), Blue color shows UTR (untranslated regions) while normal line represents introns. Figure S2. Distribution of OsS40 genes on rice chromosomes. Chromosome Map Tool was used to located genes on chromosome. Figure S3. Amino acid sequences of the four Arabidopsis, two rice and one barley protein of group I compared to the sequence of the barley HvS40 protein. The conserved DUF584 domain sequence was highlighted in black and 100% identical residues in grey. Figure S4. Conserved motifs in HvS40, AtS40–3 and OsS40 proteins. a Motif structures for the proteins were determined using MEME search tool. Grey lines represent the non-conserved sequence. Each motif is indicated by a coloered box numbered at the bottom. b Moti logo obtained by MEME program. The overall height of each stack represents the degree of conservation at each position, while the height of letters within each st [file 12870_2019_1767_MOESM2_ESM.zip › Additional file 2 Figure S8.tif]

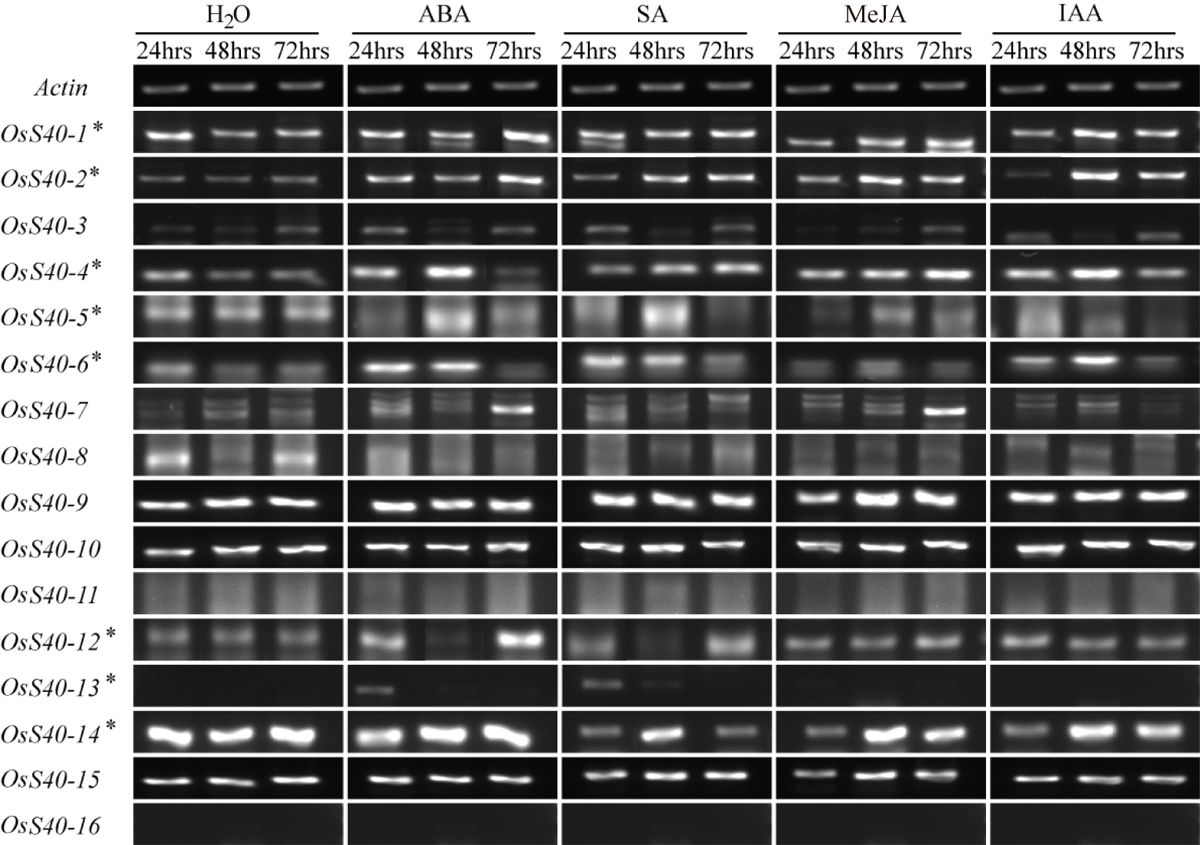

Supplement: Supplementary file 2 — Table S1. Cis elements in the promoters of S40 genes in rice, HvS40 and AtS40–3. Promoter regions of 840 bp upstream of HvS40 and 1000 bp upstream of Ats40–3, and rice S40 genes were analyzed with the use of the PLACE program. W-box: Binding site for WRKY TFs; ERE: Elicitor response element; MYB: Myeloblastosis; LREs: Light regulated elements; MYC: Myelocytomatosis; ABRE: Abscisic acid responsive elements; Dof: DNA-binding with one finger; PRE: Pathogen response elements; SURE: Sulfur response elements; DRE/CRT: Dehydration response elements/C-repeat; LTR: Low temperature response; ARF: Auxin response factor; DPBFCOREDCDC3: BZIP TFs binding core sequence; G-box plus G: TF OsIRO2-binding core sequence. Table S2. Characteristics of rice S40 proteins. Characteristics of rice S40 proteins including theoretical isoionic point (PI), molecular weight (MW), Number of amino acids, instability index, aliphatic index and GRAVY (Grand Average of Hydropathy) predicted by ProtParam tool (http://web.expasy.org/protparam/). Figure S1. Exon-intron structures of S40 genes in rice genome. Yellow color shows CDS (exon), Blue color shows UTR (untranslated regions) while normal line represents introns. Figure S2. Distribution of OsS40 genes on rice chromosomes. Chromosome Map Tool was used to located genes on chromosome. Figure S3. Amino acid sequences of the four Arabidopsis, two rice and one barley protein of group I compared to the sequence of the barley HvS40 protein. The conserved DUF584 domain sequence was highlighted in black and 100% identical residues in grey. Figure S4. Conserved motifs in HvS40, AtS40–3 and OsS40 proteins. a Motif structures for the proteins were determined using MEME search tool. Grey lines represent the non-conserved sequence. Each motif is indicated by a coloered box numbered at the bottom. b Moti logo obtained by MEME program. The overall height of each stack represents the degree of conservation at each position, while the height of letters within each st [file 12870_2019_1767_MOESM2_ESM.zip › Additional file 2 Figure S9.tif]

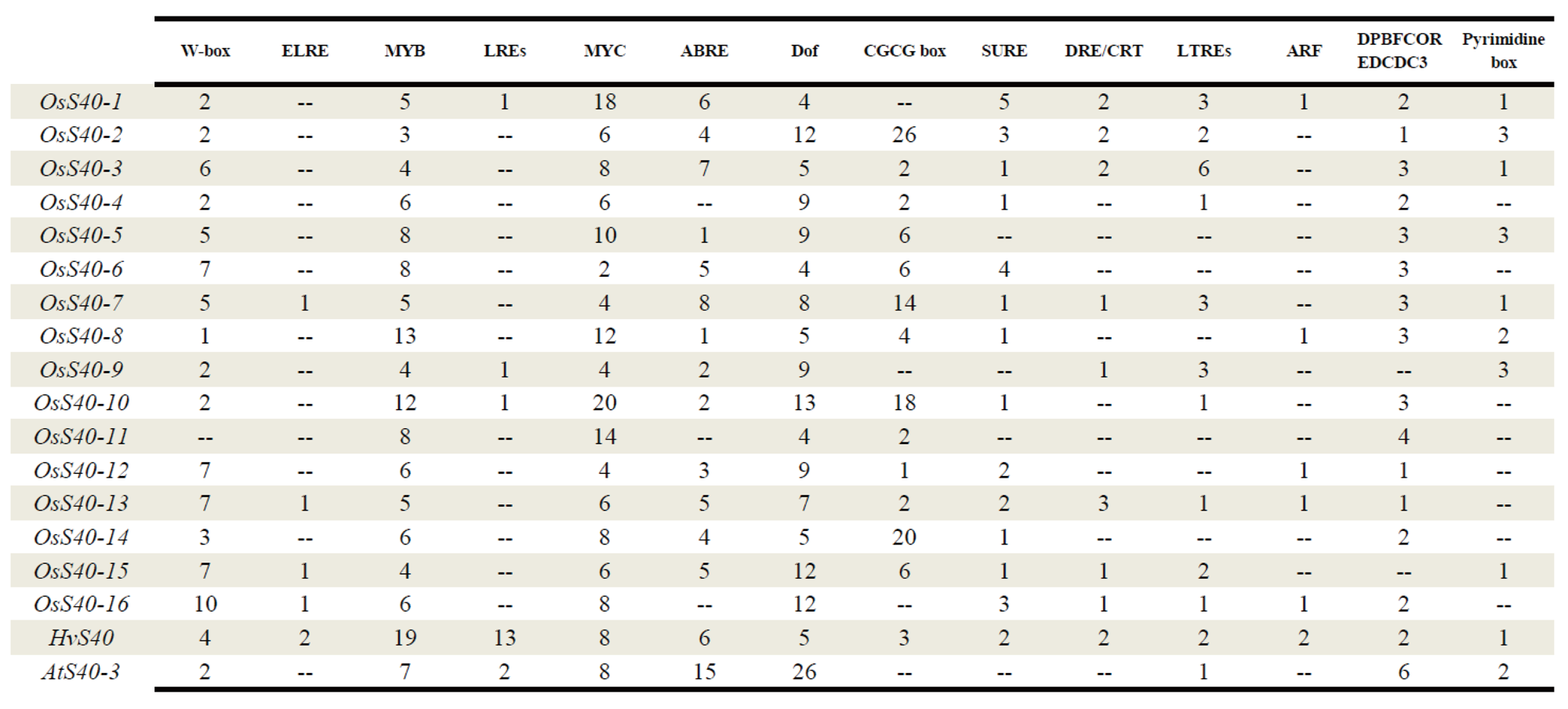

Supplement: Supplementary file 2 — Table S1. Cis elements in the promoters of S40 genes in rice, HvS40 and AtS40–3. Promoter regions of 840 bp upstream of HvS40 and 1000 bp upstream of Ats40–3, and rice S40 genes were analyzed with the use of the PLACE program. W-box: Binding site for WRKY TFs; ERE: Elicitor response element; MYB: Myeloblastosis; LREs: Light regulated elements; MYC: Myelocytomatosis; ABRE: Abscisic acid responsive elements; Dof: DNA-binding with one finger; PRE: Pathogen response elements; SURE: Sulfur response elements; DRE/CRT: Dehydration response elements/C-repeat; LTR: Low temperature response; ARF: Auxin response factor; DPBFCOREDCDC3: BZIP TFs binding core sequence; G-box plus G: TF OsIRO2-binding core sequence. Table S2. Characteristics of rice S40 proteins. Characteristics of rice S40 proteins including theoretical isoionic point (PI), molecular weight (MW), Number of amino acids, instability index, aliphatic index and GRAVY (Grand Average of Hydropathy) predicted by ProtParam tool (http://web.expasy.org/protparam/). Figure S1. Exon-intron structures of S40 genes in rice genome. Yellow color shows CDS (exon), Blue color shows UTR (untranslated regions) while normal line represents introns. Figure S2. Distribution of OsS40 genes on rice chromosomes. Chromosome Map Tool was used to located genes on chromosome. Figure S3. Amino acid sequences of the four Arabidopsis, two rice and one barley protein of group I compared to the sequence of the barley HvS40 protein. The conserved DUF584 domain sequence was highlighted in black and 100% identical residues in grey. Figure S4. Conserved motifs in HvS40, AtS40–3 and OsS40 proteins. a Motif structures for the proteins were determined using MEME search tool. Grey lines represent the non-conserved sequence. Each motif is indicated by a coloered box numbered at the bottom. b Moti logo obtained by MEME program. The overall height of each stack represents the degree of conservation at each position, while the height of letters within each st [file 12870_2019_1767_MOESM2_ESM.zip › Additional file 2 Table S1.tif]

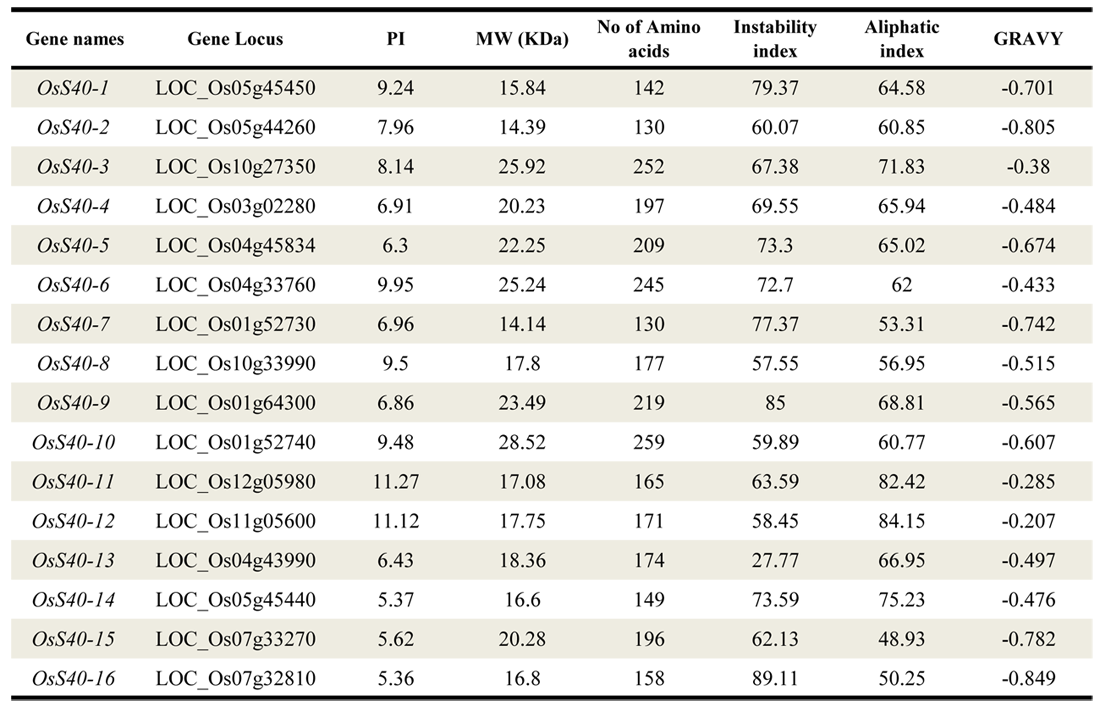

Supplement: Supplementary file 2 — Table S1. Cis elements in the promoters of S40 genes in rice, HvS40 and AtS40–3. Promoter regions of 840 bp upstream of HvS40 and 1000 bp upstream of Ats40–3, and rice S40 genes were analyzed with the use of the PLACE program. W-box: Binding site for WRKY TFs; ERE: Elicitor response element; MYB: Myeloblastosis; LREs: Light regulated elements; MYC: Myelocytomatosis; ABRE: Abscisic acid responsive elements; Dof: DNA-binding with one finger; PRE: Pathogen response elements; SURE: Sulfur response elements; DRE/CRT: Dehydration response elements/C-repeat; LTR: Low temperature response; ARF: Auxin response factor; DPBFCOREDCDC3: BZIP TFs binding core sequence; G-box plus G: TF OsIRO2-binding core sequence. Table S2. Characteristics of rice S40 proteins. Characteristics of rice S40 proteins including theoretical isoionic point (PI), molecular weight (MW), Number of amino acids, instability index, aliphatic index and GRAVY (Grand Average of Hydropathy) predicted by ProtParam tool (http://web.expasy.org/protparam/). Figure S1. Exon-intron structures of S40 genes in rice genome. Yellow color shows CDS (exon), Blue color shows UTR (untranslated regions) while normal line represents introns. Figure S2. Distribution of OsS40 genes on rice chromosomes. Chromosome Map Tool was used to located genes on chromosome. Figure S3. Amino acid sequences of the four Arabidopsis, two rice and one barley protein of group I compared to the sequence of the barley HvS40 protein. The conserved DUF584 domain sequence was highlighted in black and 100% identical residues in grey. Figure S4. Conserved motifs in HvS40, AtS40–3 and OsS40 proteins. a Motif structures for the proteins were determined using MEME search tool. Grey lines represent the non-conserved sequence. Each motif is indicated by a coloered box numbered at the bottom. b Moti logo obtained by MEME program. The overall height of each stack represents the degree of conservation at each position, while the height of letters within each st [file 12870_2019_1767_MOESM2_ESM.zip › Additional file 2 Table S2.tif]
